# Study on Printability, Mechanical Properties, and Strength Prediction of 3D-Printed Modified Recycled Micro-Powder Concrete

**DOI:** 10.3390/ma19214508

**Published:** 2026-10-26

**Authors:** Zhilin Jin, Chunyuan Wang, Ying Xu, Qiwei Zhan, Haitao Hu, Yongqi Zhao

**Affiliations:** 1 School of Civil Engineering and Architecture, Jiangsu University of Science and Technology, Zhenjiang 212000, China; 2 Zhenjiang Key Laboratory of Infrastructure Renewal and Resilience Enhancement, Zhenjiang 212000, China; 3 School of Transportation, Southeast University, Nanjing 210000, China

**Keywords:** 3D printing concrete, rheological properties, printability, mechanical properties, prediction model

## Abstract

The influence of recycled powder obtained by combined treatment of acidification and carbon activation on the printing feasibility and the mechanical properties of the material is investigated, and a predictive model for the development of mechanical properties has been developed by combining practical experiments with theoretical modeling. When the flow of the jet is between 160 mm and 170 mm, the extrusion performance of the material is optimal, allowing continuous printing of 20 layers with a stable forming height of up to 200 mm. Mechanical tests indicate a significant increase in interlayer adhesion strength, with marked anisotropy of compressive strength in different directions: Mechanical tests reveal significant anisotropy in compressive strength: the highest strength is observed in the T1 direction, followed by the T2 direction, while the T3 direction shows relatively lower strength. In a subsequent modeling analysis, three machine learning algorithms—XGBoost, Random Forest, and LightGBM—were compared in terms of compressive strength prediction. After training and validation, the XGBoost model performs the best, with MAE below 1.5 MPa and R^2^ not less than 0.91; Random Forest and LightGBM are second. Among them, LightGBM has a large prediction error in the Z direction, with a MAE of 2.30 MPa. The XGBoost model proved to be the most efficient. Thus, this study provides an effective tool for the design and regulation of 3D Printed Concrete (3DPC) properties while exploring viable paths for the recovery of construction waste in the field of advanced manufacturing, thus fostering the joint development of resource circulation and digital construction.

## 1. Introduction

As one of the core technologies driving the development of Construction Industry 4.0, 3DPC technology is profoundly transforming traditional construction methods. This technology achieves digital manufacturing of components through the layer-by-layer accumulation of materials, demonstrating significant advantages such as high design freedom, formwork-free construction, a high level of automation, and improved material utilization. It provided a new technical pathway for the construction industry to advance toward intelligent, personalized, and sustainable development [1,2,3]. In recent years, with continuous advancements in printing equipment, material systems, and process control technologies, 3DPC has shown broad application potential in fields including building construction, bridges, infrastructure, and even space construction [4,5,6].

At the same time, the rapid growth of the global construction industry has generated a large amount of solid construction waste. Taking China as an example, the annual output of construction waste has exceeded several billion tons, and the level of resource recovery urgently needs improvement [7]. Recycled Hardened Concrete Powder (RHCP) refers to powder material with particle sizes smaller than 75 microns obtained by crushing, sorting, and fine grinding discarded concrete. As a high-value-added resource-recovery method, the production volume of RHCP was enormous, but currently, much of it was treated as low-value material or directly landfilled, resulting in serious resource waste and increased environmental burden. Therefore, incorporating RHCP as a supplementary cementitious material to partially replace cement in 3DPC was regarded as a key strategy for achieving closed-loop recycling of construction waste [8,9,10]. However, RHCP itself had drawbacks such as a relatively high water absorption rate and low pozzolanic activity, and its particle surfaces were often coated with aged hydration products, such as dense C-S-H gel layers and carbonated calcium hydroxide layers [11,12,13]. If such powders were directly incorporated, the workability of 3DPC would significantly decline, strength development would be inhibited, and long-term durability may be adversely affected. Therefore, how to efficiently activate and modify RHCP to meet, or even enhance, the stringent rheological and mechanical performance requirements of 3DPC has become a core scientific issue for integrating construction solid-waste recycling with advanced intelligent manufacturing technologies and promoting the development of green, low-carbon 3D printing technology. To address these challenges, this study attempted to introduce acidified and carbonated modified blast furnace slag powder (CA-RHCP) as a novel cementitious material to partially replace cement in 3DPC [14,15]. The core objective of the modification was to stimulate the latent cementitious activity of RHCP and optimize its particle surface characteristics to adapt to and improve the overall performance of 3DPC. Experimental results showed that after acidification and carbonation treatment, the specific surface area of RHCP significantly increased, water absorption decreased, and both its pozzolanic and micro-aggregate-filling effects were simultaneously enhanced.

However, as an additive manufacturing technology, 3DCP posed unprecedented challenges to the performance of concrete materials. The key lay in the material needing to possess two seemingly contradictory rheological properties: during pumping and extrusion, the material must have a relatively low dynamic yield stress to ensure it could smoothly pass through the delivery pipeline and nozzle, achieving good extrudability; yet after extrusion and deposition, the material must quickly recover and maintain a high static yield stress to resist its own weight and the load from upper layers, ensuring the geometric accuracy of the component and preventing collapse or deformation. This property was known as buildability or shape stability [16,17,18]. This typical “shear-thinning” behavior, coupled with rapid structural recovery, was a prerequisite for high-quality 3D printing [19]. Furthermore, as digital manufacturing evolved from prototyping to full-scale construction, 3D concrete printing was revolutionizing construction methodologies through its formless production approach [20,21]. The material’s shear-thinning behavior and rapid structural recovery capability form the foundation for achieving high-quality 3D printing. As digital manufacturing shifts from prototyping to the production of full-scale building components, 3DPC, with its advantage of formwork-free shaping, is reshaping the traditional logic of construction, making the realization of complex geometries possible and driving the construction industry toward automation and sustainable development [22,23]. Compared to traditional monolithic cast concrete, 3DPC components exhibited significant mechanical anisotropy. Specifically, the strength along the Z-axis, perpendicular to the printing direction (hereafter referred to as the T1 direction), was mainly influenced by the interlayer bonding strength. Its compressive strength was generally higher than that in the X-axis (T2 direction) and Y-axis (T3 direction) within the printing plane [24,25,26], with the T2 direction strength being intermediate and the T3 direction the lowest. The overall structural integrity, load-bearing capacity, and long-term durability of components were significantly constrained by the quality of the interlayer interface. Weak interlayer bonding caused the mechanical performance of the component to be primarily determined by the properties of the interface rather than the bulk material. Microstructural analysis indicated that printing paths and deposition parameters had a significant impact on the quality of the interlayer transition zone, which in turn led to differences in mechanical responses in different directions. To accurately characterize such heterogeneity, various testing methods and numerical simulation techniques had been developed to quantify the mechanical behavior of components under both in-plane and out-of-plane loading conditions. Printing speed and layer thickness parameters directly affected mechanical interlocking between layers, thereby determining the final bonding strength, highlighting the necessity of synergistic optimization of process parameters and material properties [27]. This also represented a key technical bottleneck limiting the large-scale application of 3DPC in load-bearing structures [28,29].

The mechanical anisotropy of 3DPC is not a monolithic phenomenon but a process- and definition-dependent one. While a directional hierarchy of in-plane strength > interlayer strength is broadly accepted, the reported magnitude of anisotropy varies substantially across studies, ranging from roughly 20% to 50% depending on mix design, interlayer interval, and the convention used to label loading directions. This variability is not merely experimental scatter; it reflects the fact that interlayer interfaces are simultaneously mechanical weak links, transport pathways, and microstructural discontinuities whose properties are governed by the coupled effects of material rheology, deposition timing, and surface moisture state. Reviews of interlayer bonding strategies have cataloged reinforcement integration, surface treatment, nozzle modification, and mix adjustment as discrete interventions, yet they also reveal that the effectiveness of each strategy depends on whether it bridges the interlayer region rather than merely reinforcing the filament core. For powdered materials, the situation is further complicated by the fact that nucleation effects and filler effects operate at different length scales and may influence bulk matrix strength and interlayer adhesion in opposite directions. It is precisely this coupling—between a modified powder‘s surface chemistry, its rheological consequences, and its differential impact on bulk versus interfacial strength—that remains insufficiently examined in the 3DPC literature, and that motivates the present study.

This study systematically evaluated the applicability of CA-RHCP in 3DPC and its reinforcement mechanisms, with a focus on its regulatory effect on printing performance at multiple scales. In terms of printability, incorporating CA-RHCP was expected to improve the shear-thinning behavior and structural recovery rate of the paste, thereby ensuring good extrudability while enhancing shape stability and buildable height [30] after deposition. Regarding mechanical properties, CA-RHCP not only participated in hydration reactions through its active mineral phases to generate additional C-S-H gel that strengthens the matrix, but more importantly, the nanoscale calcium carbonate (CaCO_3_) attached to its surface acted as nucleation sites, promoting dense deposition of hydration products at the interlayer interface. This significantly enhanced interlayer bonding strength and mitigated the inherent anisotropy of printed components. Case analyses indicated that the performance of 3DPC was influenced by the nonlinear coupling of various mix design and process parameters, making traditional trial-and-error methods inefficient for optimization. In recent years, machine learning algorithms such as Random Forest and Extreme Gradient Boosting (XGBoost), known for their excellent nonlinear fitting capabilities [31], have been widely applied in simulating concrete strength evolution and predicting complex rheological responses. Based on these data-driven models, precise mapping from material composition to multidimensional mechanical properties could be achieved, providing effective tools for rapid and reliable quantitative design of complex systems. This method formed the core logic of this study in constructing a predictive model to optimize the performance of CA-RHCP-modified 3DPC. By introducing CA-RHCP as a functional admixture into the 3DPC system, this study aimed to provide a practical material path for the high-value utilization of construction solid waste and the green and high-performance development of 3DPC.

Previous studies in this field have provided valuable quantitative benchmarks. It has been widely reported that printable cementitious materials generally require a flow table spread of approximately 160–180 mm to ensure stable extrusion, and that properly controlled rheology allows continuous printing of more than 20 layers. However, interlayer bonding remains the weakest link in 3D printed concrete. The interlayer adhesion strength is often 20–40% lower than the bulk matrix strength, and this weak interface leads to pronounced mechanical anisotropy, with directional differences in compressive strength commonly reaching 10–30%. For recycled concrete powder, chemical activation has been shown to effectively increase pozzolanic reactivity, but unmodified recycled hardened concrete powder still causes a 15–30% reduction in 28-day strength when the replacement level exceeds 10–15%, mainly due to its high water absorption and low activity. In the field of machine learning, data-driven models have predicted the compressive strength of 3D-printed concrete with a mean absolute error of approximately 1.5–3.0 MPa and a coefficient of determination above 0.9. Despite these advances, few studies have combined acidification and carbonation activation of recycled hardened concrete powder with 3D-printed concrete, and the coupled effects of such modified powder on printability, interlayer bonding, anisotropy, and strength prediction remain unclear. To address this gap, CA-RHCP with a specific surface area of 489 m^2^/kg and approximately 58% reduced water absorption is introduced into 3DPC in this study. At a 10% replacement level, the flow spread remains 160–170 mm, 20 layers can be continuously printed to a stable height of 200 mm, and the anisotropic compressive strength follows T1 > T2 > T3. An XGBoost model predicts the compressive strength with MAE < 1.5 MPa and R^2^ ≥ 0.91, providing a reliable data-driven tool for the high-value utilization of construction waste in 3DPC.

## 2. Materials and Methods

### 2.1. Raw Materials

The raw materials used in this study include: P.O 42.5 ordinary Portland cement (specific surface area 352 m^2^/kg); CA-RHCP (specific surface area 489 m^2^/kg, with approximately 58% reduced water absorption); natural sand with particle size 0.08–2.0 mm (fineness modulus 2.3); polycarboxylate superplasticizer (water reduction rate ≥ 30%); hydroxypropyl methylcellulose thickener (viscosity 200,000 MPa·s); and polypropylene fibers with a length of 6 mm. All materials comply with relevant standards and have undergone rigorous testing. In this study, P.O. 42.5 ordinary Portland cement produced by Jiangsu Zhenjiang Helin Cement Co., Ltd., China, was used. CA-RHCP was obtained from waste concrete collected by the laboratory, which was then crushed and modified in the laboratory. The natural sand was procured through the cement plant. The polycarboxylate superplasticizer, hydroxypropyl methylcellulose thickener, and polypropylene fibers were all supplied by Hebei Fu Qiang Chemical Group, China. The production process of CA-RHCP involves crushing waste concrete test blocks into small pieces, ball milling for 30 min, sieving through a 200 mesh sieve to obtain regenerated micro powder, then using cultured lactic acid bacteria (pH 3.5) for acidification treatment, and finally placing them in a carbonization box (CO_2_ concentration of 20%, humidity of 90%, temperature of 20 °C) for carbonization treatment.

### 2.2. Printing Mix Ratio

The parameter design of this experiment adopts a five-level and six-factor parameter design to systematically study six key variables: CA-RHCP substitution rate (0%,5%,10%,15%,20%), water-to-binder ratio (0.36–0.4), bone-to-binder ratio (0.8–1.6), superplasticizer dosage (0.00–0.48%), thickener dosage (0.000–0.500%), and fiber content (0.0–0.4%). A total of 25 mix formulations were designed, with three replicates prepared for each. Preliminary experiments were conducted to adjust the water-to-binder ratio and admixture dosage to ensure comparable initial squeezability across all formulations. Printing was performed using (China Hangzhou Guanli Co., Ltd.) a laboratory-grade plunger extrusion 3D printer (model: CONPRINT 3D) with a nozzle diameter of 25 mm, layer height of 10 mm, and printing speed of 40 mm/s. Each parameter is pre-set with a base value, and when the experiment reaches the influence of a certain parameter variable, other parameters are assumed to have this base value. The basic values we have selected are a substitution amount of 10%, a water-cement ratio of 0.38, a bone cement ratio of 1.2, a thickening agent dosage of 0.25%, a water-reducing agent dosage of 0.24%, and a fiber volume fraction of 2%. For example, when verifying the effect of substitution amount on the experiment, other conditions were a water-cement ratio of 0.38, a bone cement ratio of 1.2, a thickening agent dosage of 0.25%, a water-reducing agent dosage of 0.24%, and a fiber volume fraction of 2%. All specimens were cured under standard conditions (temperature 20 ± 2 °C, humidity ≥ 95%) until the specified age for testing. The specific data for each designed mix are presented in Table 1 below:

### 2.3. Test Methods

#### 2.3.1. Printability Evaluation

To comprehensively evaluate the printability of 3D-printed concrete, this study conducted integrated assessments through Squeezability tests, buildability tests, and flowability tests. The Squeezability test utilized a self-developed piston extrusion device in the laboratory, recording the mass flow rate (g/s) over 60 s under a constant extrusion pressure of 0.5 MPa to quantify the material’s pumping and extrusion performance. Simultaneously, the continuity of the extrusion process and the surface quality of the extruded strips were observed. The buildability test involved continuous printing of 20 layers of wall structures under standard printing parameters: 10 mm layer height and 40 mm/s printing speed. After printing, the structures were left undisturbed for 5 min, with measurements taken for maximum collapse-free height and horizontal deformation of each layer to evaluate the material’s shape retention capability and structural stability post-extrusion. Additionally, a standard flowability test (per ASTM C1437 [32]) was conducted to measure the initial flow diameter of fresh concrete slurry, serving as a fundamental indicator for evaluating the material’s workability.

#### 2.3.2. Mechanical Properties and Microstructural Analysis

To comprehensively evaluate the anisotropic mechanical properties of 3D-printed components, various types of specimens were fabricated and tested according to the printing-direction system. The testing instrument used is the TYE-300D cement mortar flexural and compressive testing machine produced by Jinan Yinuo Testing Instrument Factory, Jinan, China. The compressive strength tests were conducted in accordance with the JGJ/T 70-2009 standard [33], with 70 mm cubic specimens prepared and tested in both the direction parallel to the printing layer (T2/T3 direction) and the direction perpendicular to the printing layer (T1 direction), at a loading rate of 0.6 MPa/s.

Characterizing the microstructure may involve three components. By employing multi-scale microstructural analysis techniques, a systematic study of the material’s composition and structural evolution and their correlations with macroscopic properties is conducted.

Optical microscope: A stereoscopic optical microscope (Olympus SZ61, Olympus Corporation, Tokyo, Japan) is used for macroscopic observation and low-magnification examination of the surface morphology of printed ribbons and interlayer interfaces. After natural drying, samples are placed directly on the work platform, and by adjusting the depth of field and light source angle, the uniformity of extrusion, surface texture, layer-to-layer overlap, as well as macroscopic defects (such as cracks or voids) are recorded and analyzed. This observation aims to assess the quality of printing at a mesoscale level, visually reflecting the material’s ability to maintain its shape and overall bonding integrity during the deposition process, thereby providing a foundation for subsequent high-resolution microscopic analysis.

Scanning electron microscope: A field-emission scanning electron microscope (FE-SEM; the Hitachi SU5000, Hitachi High-Technologies Corporation, Tokyo, Japan) is used to obtain high-resolution images of the fresh fracture surfaces of printed ribbons, the interlayer transition zones, and hydrated products of different ages. The samples are examined after vacuum drying and gold-deposition treatment, with an acceleration voltage of 5–15 kV. This analysis aims to directly visualize microstructural features, such as the morphology of CaCO_3_ deposits, the structure of the C-S-H gel, pore distribution, and fiber-matrix bonding conditions.

BET analysis between layers: A printing sample from the interlayer transition zone is taken as raw material for testing the BET specific surface area. A specific surface area and pore size analyzer (Micromeritics ASAP 2460, Micromeritics Instrument Corporation, Norcross, GA, USA) is used, applying the low-temperature nitrogen adsorption method to quantitatively characterize the porous structure of the powder sample. After pretreatment by vacuum degassing at 60 °C, the sample undergoes isothermal nitrogen adsorption–desorption testing at 77 K, and the specific surface area is calculated using the BET (Brunauer–Emmett–Teller) model. The mesoporous distribution (2–50 nm) is analyzed using the BJH (Barrett-Joyner-Halenda) model, while the overall pore distribution is evaluated using the DFT (density functional theory) model. This analysis aims to quantitatively determine the pore volume, specific surface area, and pore size distribution within the material, with a particular focus on the evolution of the porous structure in the interlayer transition zone, to clarify the mechanism of compaction in this region due to CA-RHCP modification.

#### 2.3.3. Model Prediction

This study systematically compares three representative algorithms: XGBoost, an extreme gradient boosting tree renowned for its robust handling of nonlinear relationships and high precision; LightGBM, an efficient gradient boosting-based algorithm particularly suited for datasets with high feature dimensions; and Random Forest, whose core mechanism and advantages as an ensemble learning algorithm are highlighted. By analyzing the predictive accuracy and generalization capabilities of these models for 3DPC compressive strength, we aim to establish a reliable and efficient strength-prediction tool. This work provides data-driven theoretical foundations and intelligent solutions for 3DPC material design and process parameter optimization based on CA-RHCP, ultimately laying a solid theoretical and technical foundation for the large-scale, high-value utilization of construction solid waste in advanced intelligent manufacturing [34,35]. Using 5-fold cross-validation and grid search for hyperparameter optimization, the data is divided into 80% training and 20% testing. Main hyperparameter range: XGBoost hyperparameter range: n_estimators, max_depth, learning_rate, subsample, colsample_bytree, reg_alpha, reg_lambda; Random Forest hyperparameter range: n_estimators, max_depth, min_samples_split, min_samples_leaf, max_features; LightGBM hyperparameter range: num_leaves, max_depth, learning_rate, n_estimators, min_child_samples, subsample, colsample_bytree.

## 3. Results

### 3.1. Effect of CA-RHCP on 3DPC Printability

**Figure 1:**
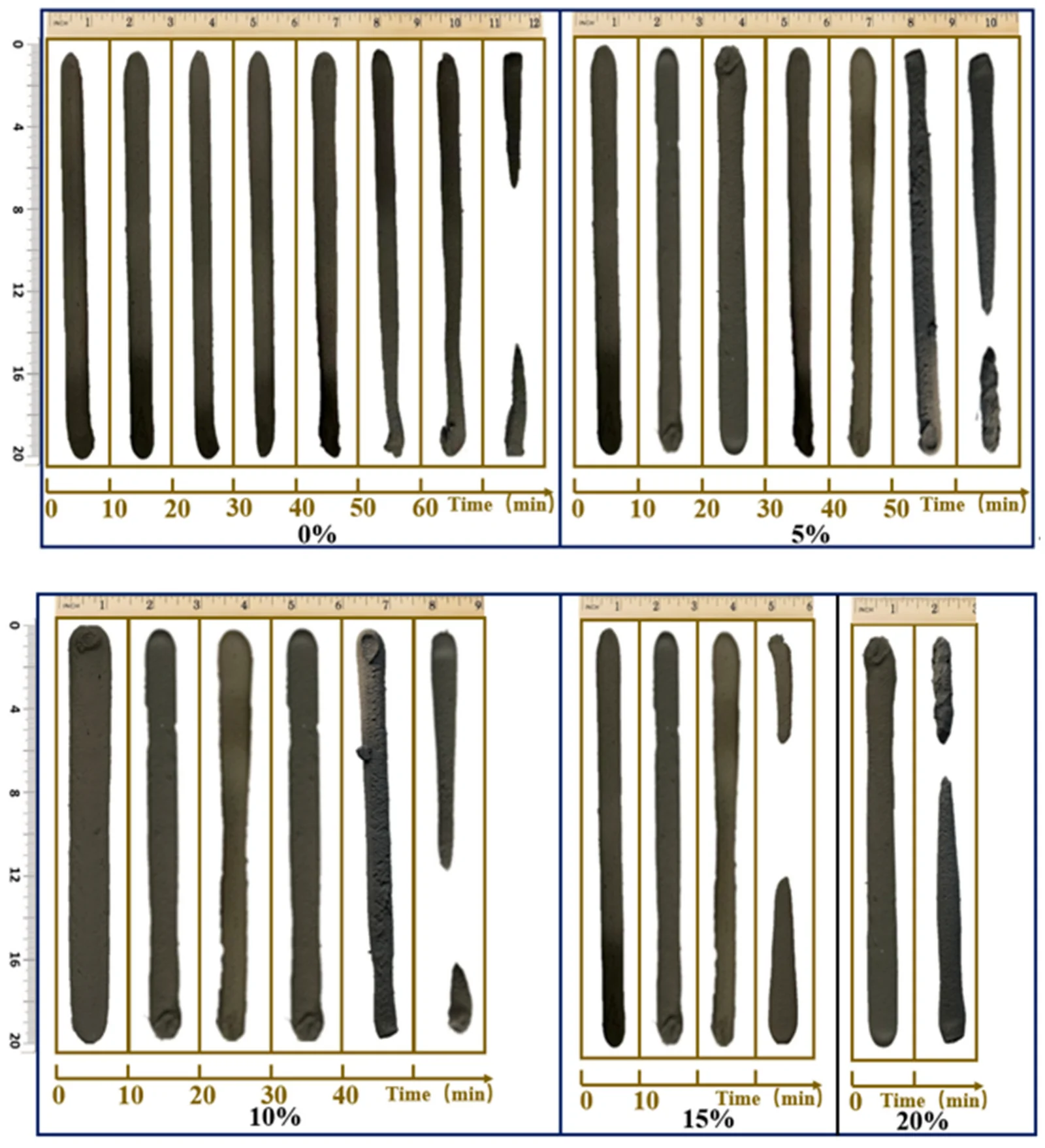
Print strip diagram under various substitution conditions.

From Figure 1, The strip chart, open time, and flowability histogram demonstrate that optimal printability is achieved at 0% substitution. This occurs because the cement’s activity exceeds that of the CA-RHCP cementitious material being substituted. As substitution increases, the strip’s open time progressively decreases, indicating that reduced active components lead to higher water absorption and shorter open times. At 20% substitution, the open time reaches zero, confirming that the substitution limit is 20%. Subsequent substitutions are uniformly set at 10%.

**Figure 2:**
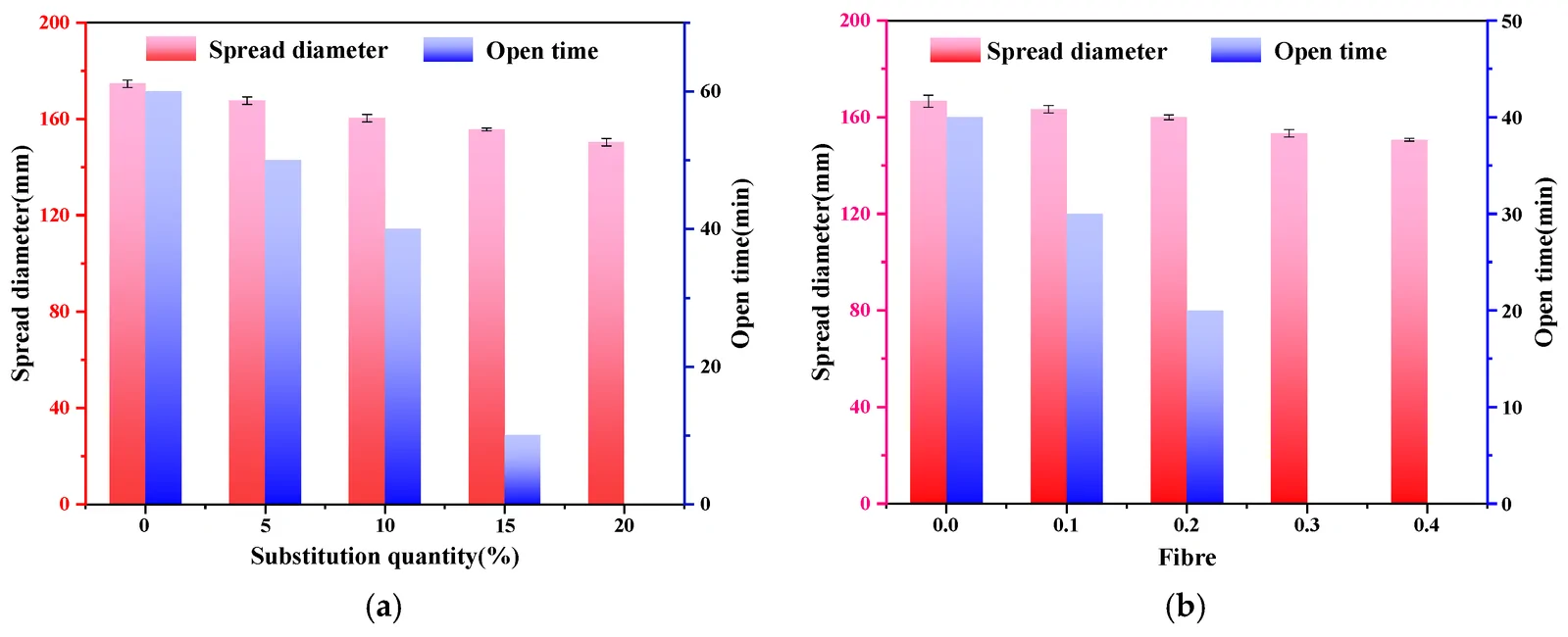

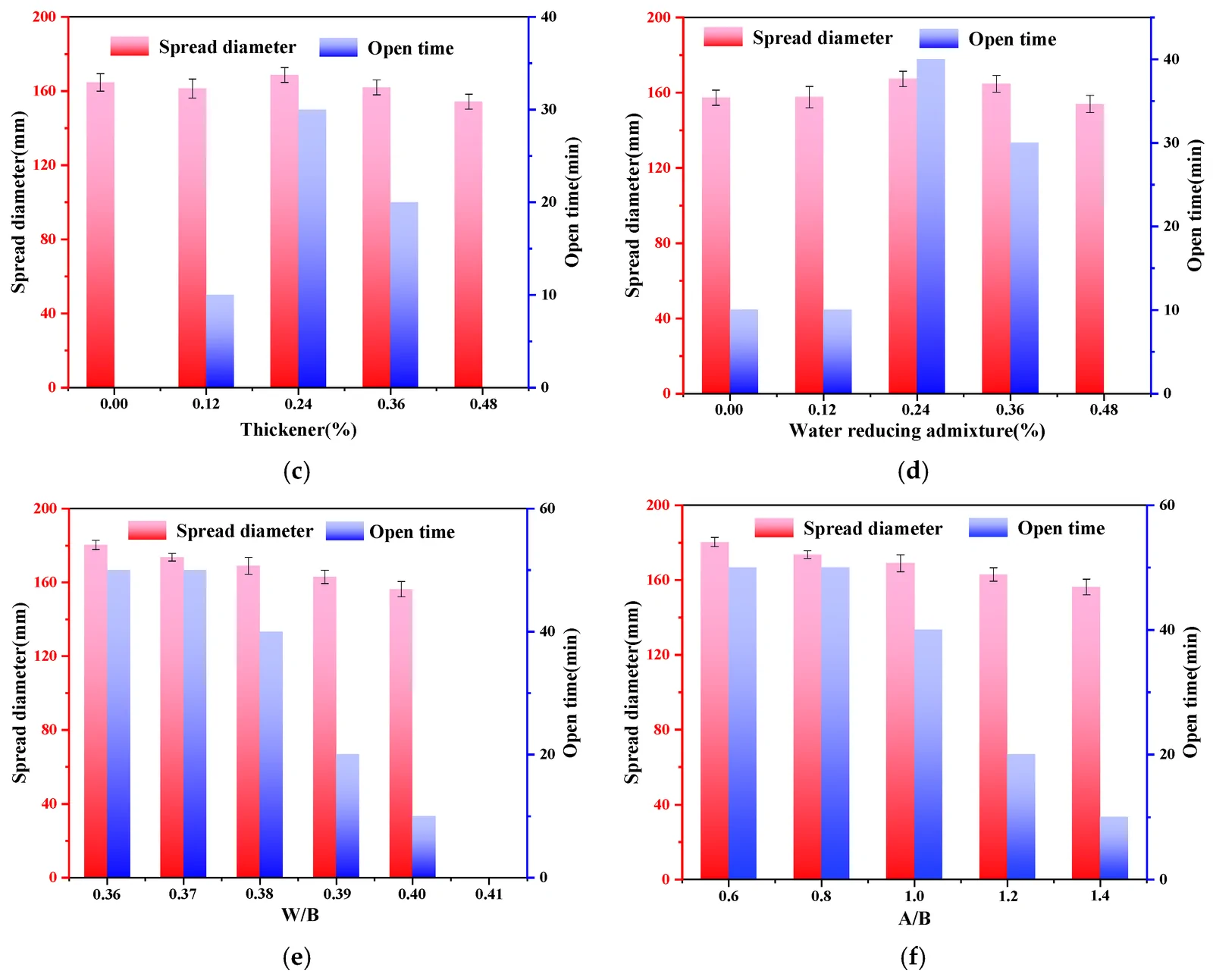
Flowability and open time of 3DPC under various parameter conditions: (**a**) Substitute quantity; (**b**) Fiber content; (**c**) Thickener; (**d**) Water reducer; (**e**) Water-to-bonding agent ratio; (**f**) Bone-to-bonding agent ratio.

Figure 2 illustrates the relationship between the proportion parameters (fiber, thickener, water-reducing admixture, water-cement ratio, and cement-water ratio) and the operational performance of the 3DPC, including fluidity and setting time. It can be observed that fluidity and setting time exhibit a balanced relationship, where one affects the other. For example, as seen in Figure 2d,e, increasing the water-reducing admixture or the water-cement ratio significantly improves fluidity but adversely affects setting time and form stability. Conversely, as shown in Figure 2b,c, increasing the thickener or fiber enhances the structural strength of the system, prolonging setting time, but reduces fluidity and increases extrusion resistance. Figure 2f indicates that increasing the cement-water ratio results in a reduction in the relative water content in the mortar, reinforcing the structural role of the aggregates, while also decreasing fluidity and promoting better form retention. These results indicate that achieving excellent impression quality requires coordinated optimization of multiple parameters, aiming for the ideal balance between “extrudability” and “construction capacity.”

The bandwidth, open time, and flow of the table can not only indicate the quality of the print performance, but also the key parameters of the constructability.

**Figure 3:**
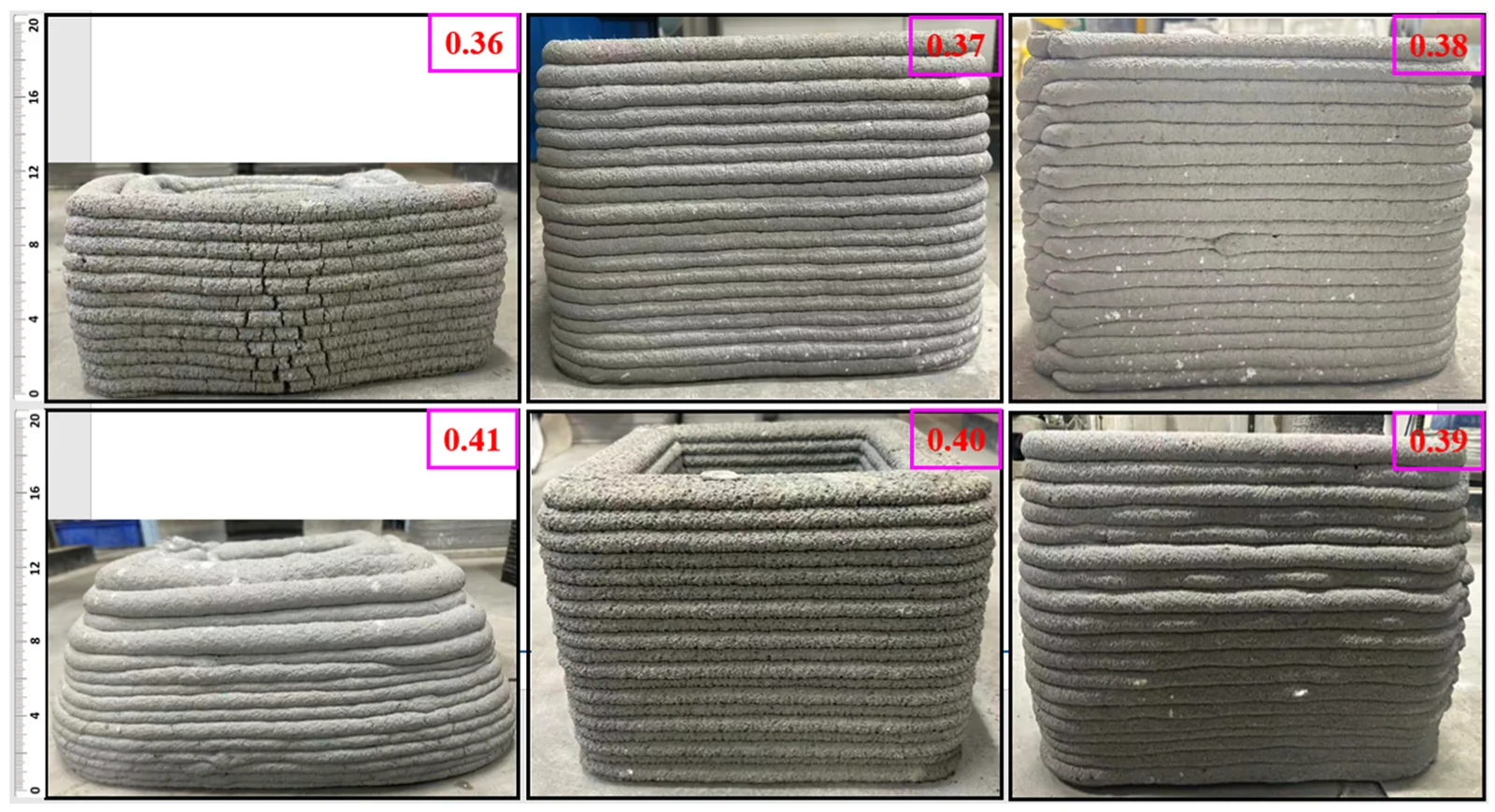
Comparison of constructability of printed components under different water-to-binder ratios.

In the construction experiment, the buildability varied under different parameter conditions, most visibly reflected in differences in build height. As a key metric for evaluating 3DPC’s printability, this study defined concrete with 3DPC exhibiting good printability as having a continuous print height of no less than 200 mm. From Figure 3, Taking the water-to-binder ratio as an example, a ratio of 0.36 resulted in poor buildability, with the build height failing to reach 200 mm and interlayer cracking and collapse occurring. This was attributed to the excessively low water-to-binder ratio causing the printed material to become overly dry, thereby compromising buildability. Similarly, a ratio of 0.41 also failed to achieve the 200 mm build height, while an excessively high ratio led to noticeable sagging in the printed structure. All other parameter groups maintained excellent buildability, consistently reaching the 200 mm target at a print height of 20 layers. These test specimens across all parameter groups demonstrate superior buildability performance.

**Figure 4:**
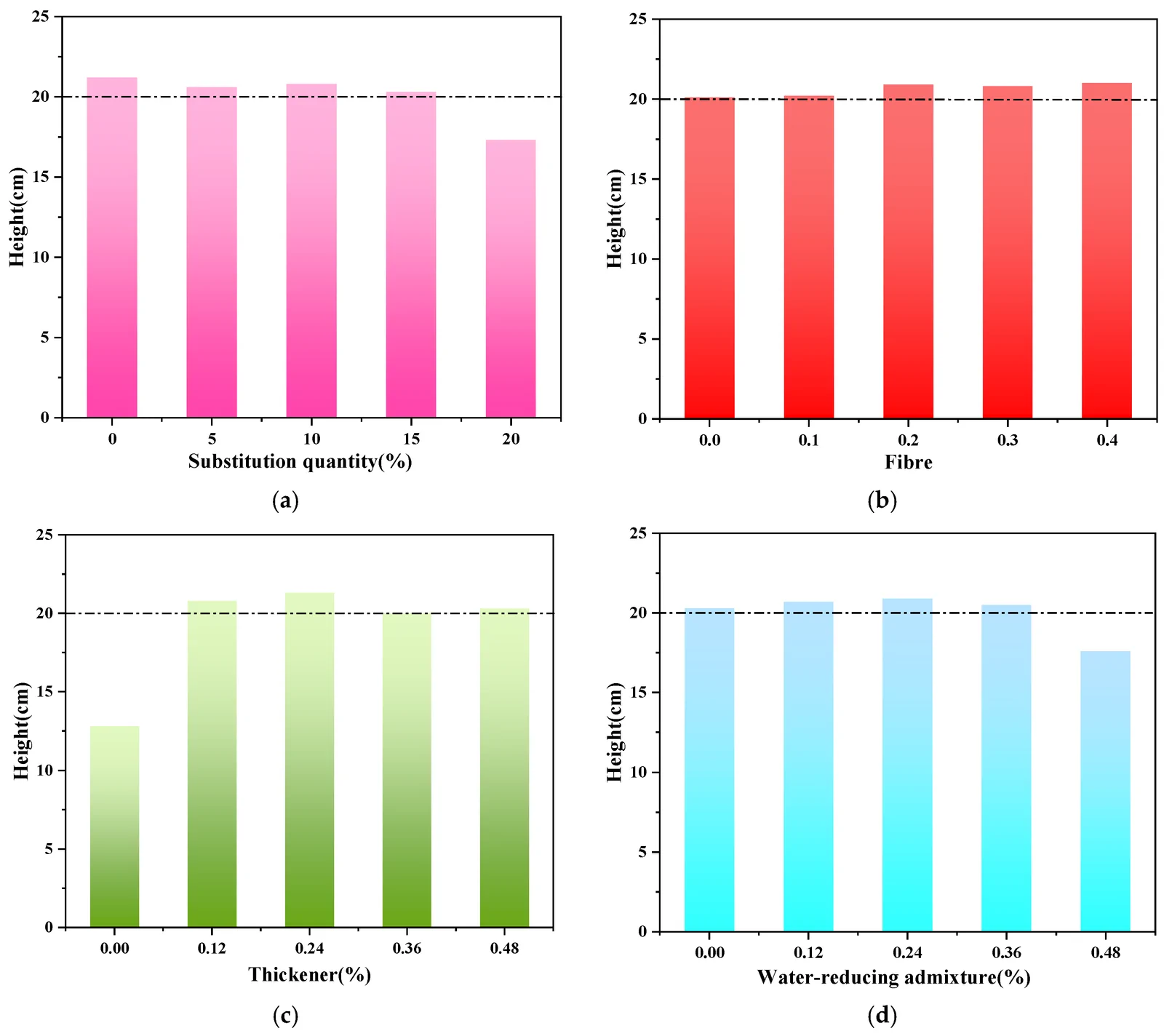

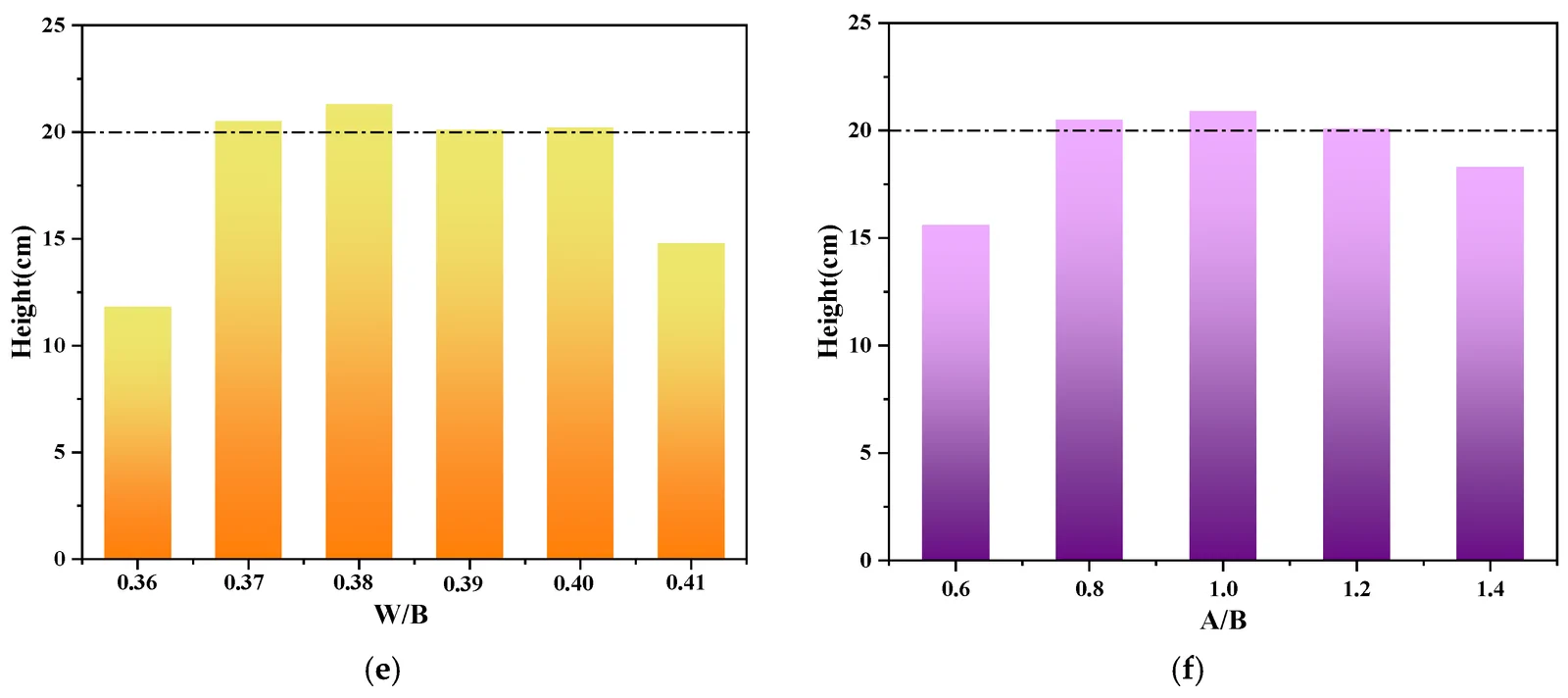
Comparison of construct height under different parameter conditions: (**a**) substitution amount; (**b**) fiber content; (**c**) thickener; (**d**) superplasticizer; (**e**) water-to-binder ratio; (**f**) bone-to-binder ratio.

The constructability height comparison in Figure 4 demonstrates that excessive substitution volume and superplasticizer dosage result in failure to meet standard requirements. Conversely, insufficient thickener dosage may also prevent the construction height from reaching 200 mm. Both excessively high and low water-to-binder ratios (W/B) and bone-to-binder ratios (B/B) negatively impact constructability, ultimately preventing the construction height from achieving the 200 mm target. Among all variables, fiber dosage has the least significant effect on constructability.

Construction performance is primarily governed by the rheological behavior of the slurry, with various mix design parameters influencing structural formation and stability through distinct mechanisms. When the replacement ratio of CA-RHCP exceeds 15%, the abundant low-reactivity particles and their high specific surface area adsorb excessive water, resulting in insufficient free water for hydration. This delays early structural formation and the growth of static yield stress, thereby compromising the material’s deformation resistance and collapse resistance. When the superplasticizer content surpasses 0.36%, it excessively disperses particles, disrupts the slurry’s flocculation structure, and delays thixotropic recovery, hindering the sediment layer from rapidly achieving the required strength to support the upper layer. Conversely, when the thickener content falls below 0.125%, the slurry lacks adequate cohesion and water retention, leading to water bleeding and particle settling that damages interlayer bonding and overall stability. Deviations in the water-to-binder ratio also adversely affect construction: excessively high ratios result in low slurry viscosity, causing flowability issues, while excessively low ratios impair extrusion and interlayer fusion, making it difficult to form uniform and stable sediment layers. Imbalanced binder-to-aggregate ratios similarly impact construction—excessively low ratios increase shrinkage due to excess slurry, while excessively high ratios fail to adequately encapsulate aggregates, increasing friction and pore formation, both of which reduce the integrity and self-supporting capacity of printed components. Notably, fiber content within the test range (≤0.4%) has minimal effect on construction height, primarily because the short-cut polypropylene fibers (6 mm) in fresh slurry mainly provide subtle network support, with their impact on rheological properties being far less pronounced than their enhancement of mechanical properties post-hardening. Therefore, to achieve good construction performance, it is necessary to optimize each parameter systematically to ensure that the material has rapid structural recovery capability and sufficient shape stability after extrusion.

### 3.2. Effect of CA-RHCP on Mechanical Properties and Microstructure

#### 3.2.1. Anisotropic Mechanical Properties of 3DPC Concrete Test Blocks

**Figure 5:**
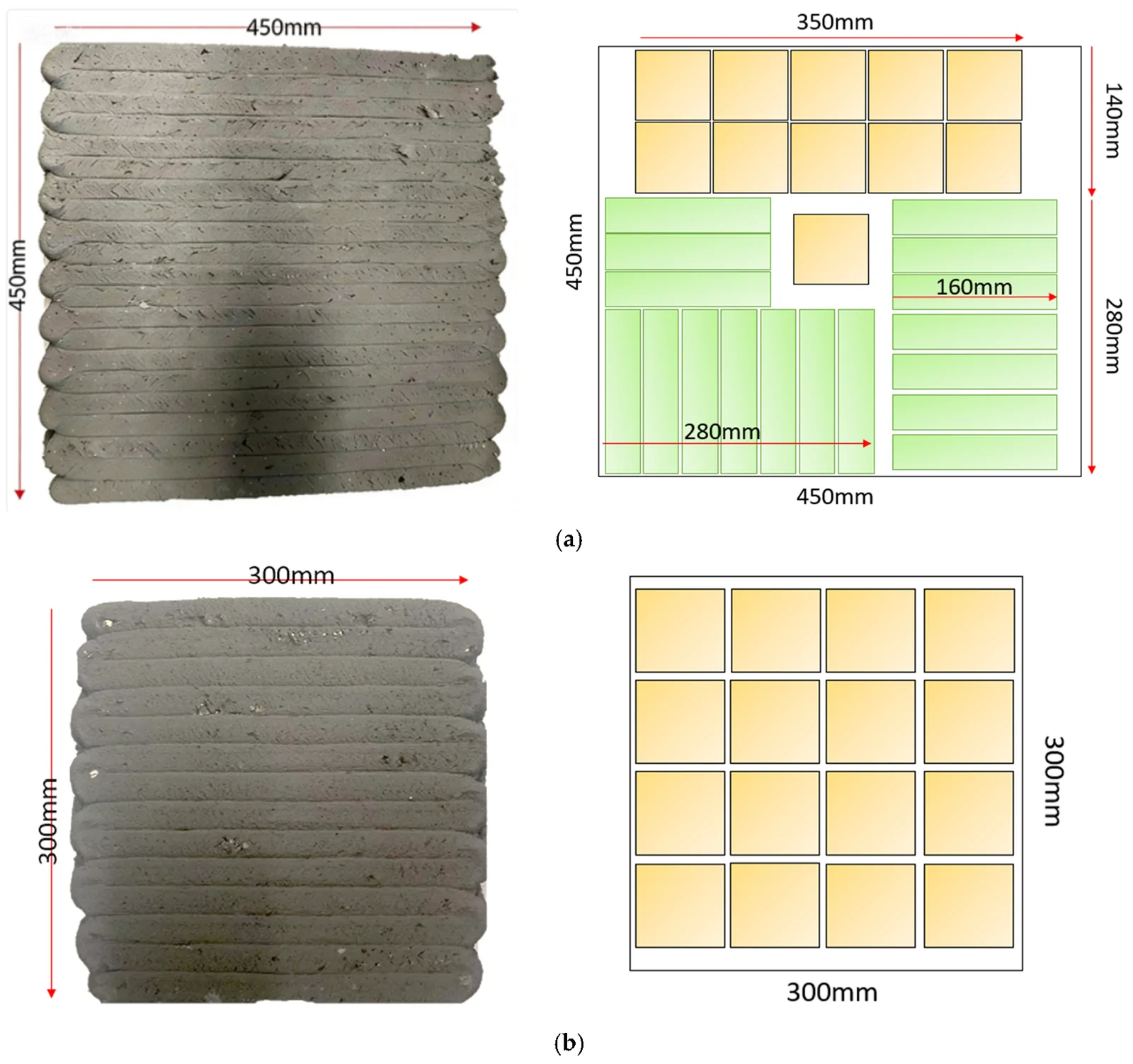
3DPC component diagram and block cutting schematic: (**a**) 450 mm × 450 mm; (**b**) 300 mm × 300 mm.

As shown in Figure 5, the experimental components consist of a large rectangular specimen (450 mm × 450 mm) and a medium-sized specimen (300 mm × 300 mm), both with a height of 100 mm. To conduct subsequent compressive and flexural strength tests, these specimens require cutting. The cutting process yields 27 cubic compressive specimens (70 mm × 70 mm × 70 mm), corresponding to 9 specimens per direction. Additionally, 18 flexural specimens (40 mm × 40 mm × 160 mm) are produced for testing in the X and Y directions. The experiment designs 25 parameter sets, generating 675 compressive test data points in total, with 225 data points per direction across the X, Y, and Z axes. For flexural strength testing, 450 sets of data are collected in the XY directions, each containing 225 test samples, which will be used for predictive modeling in subsequent experiments.

**Figure 6:**
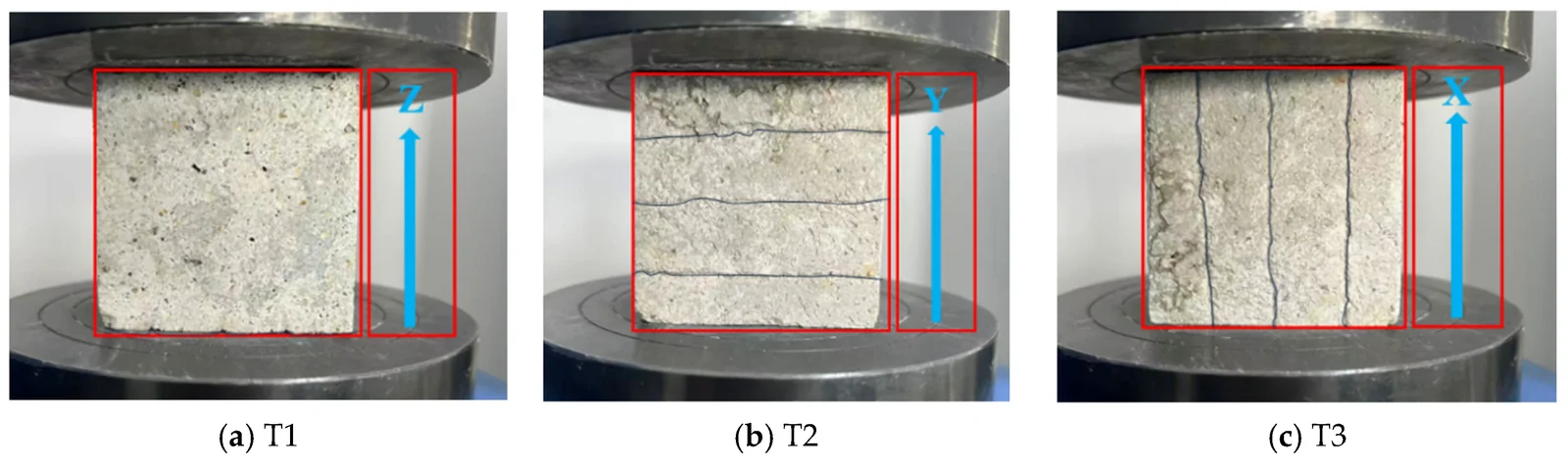
Schematic diagram of 3DPC loading. (**a**) T1 direction (**b**) T2 direction (**c**) T3 direction.

As shown in Figure 6, the 3DPC sample is divided into three directions based on the printing process: the vertical layer-by-layer stacking direction (Z direction, T1), the horizontal direction perpendicular to the printing path (Y direction, T2), and the X direction along the printing path (T3) [36]. A note on direction conventions is warranted before comparing these results with the broader literature. In the present study, T1 denotes the direction perpendicular to the interlayer plane, T2 the direction within the printing plane but perpendicular to the filament path, and T3 the direction along the filament path. Many studies report a hierarchy of X > Y > Z, where X is the filament direction, Y the inter-filament direction within the plane, and Z the interlayer direction. The present T1 > T2 > T3 order is therefore not a contradiction of the X > Y > Z convention; it reflects a different but internally consistent directional nomenclature. What matters for cross-study comparison is the relative magnitude of the interlayer direction. Here the T1-to-T3 difference is approximately 20–25% at the 10% substitution level, which falls within but toward the lower bound of the 20–50% range reported in statistical compilations of published 3DPC data [37]. This relatively moderate anisotropy is consistent with the intended function of CA-RHCP: the fine carbonated particles densify the interlayer transition zone without disrupting the continuity of the bulk matrix, so the interface does not degrade to the extent observed in mixtures where interlayer bonding is neglected.

As detailed in Section 3.2, the printability of materials is significantly influenced by the substitution ratio. With increasing substitution, the 3D-printed concrete (3DPC) specimens demonstrate progressively reduced printability, characterized by shortened open times and a substantial decrease in flowability. This trend is also reflected in the mechanical properties. The strength results under various substitution ratios shown in Figure 5 indicate a negative correlation between substitution ratio and both compressive and flexural strengths. For instance, when the substitution ratio reaches 20%, the compressive and flexural strengths in all directions are lower than those of the unsubstituted control group. Notably, the reduction in compressive strength in the T1 direction is particularly pronounced. This is primarily attributed to the lower reactivity of CA-RHCP compared to cement, resulting in insufficient hydration of the substituted component. Consequently, the formation of dense hydration product structures is inadequate, leading to insufficient micro-density in the interlayer regions of the printed layer, which significantly weakens the compressive strength.

**Figure 7:**
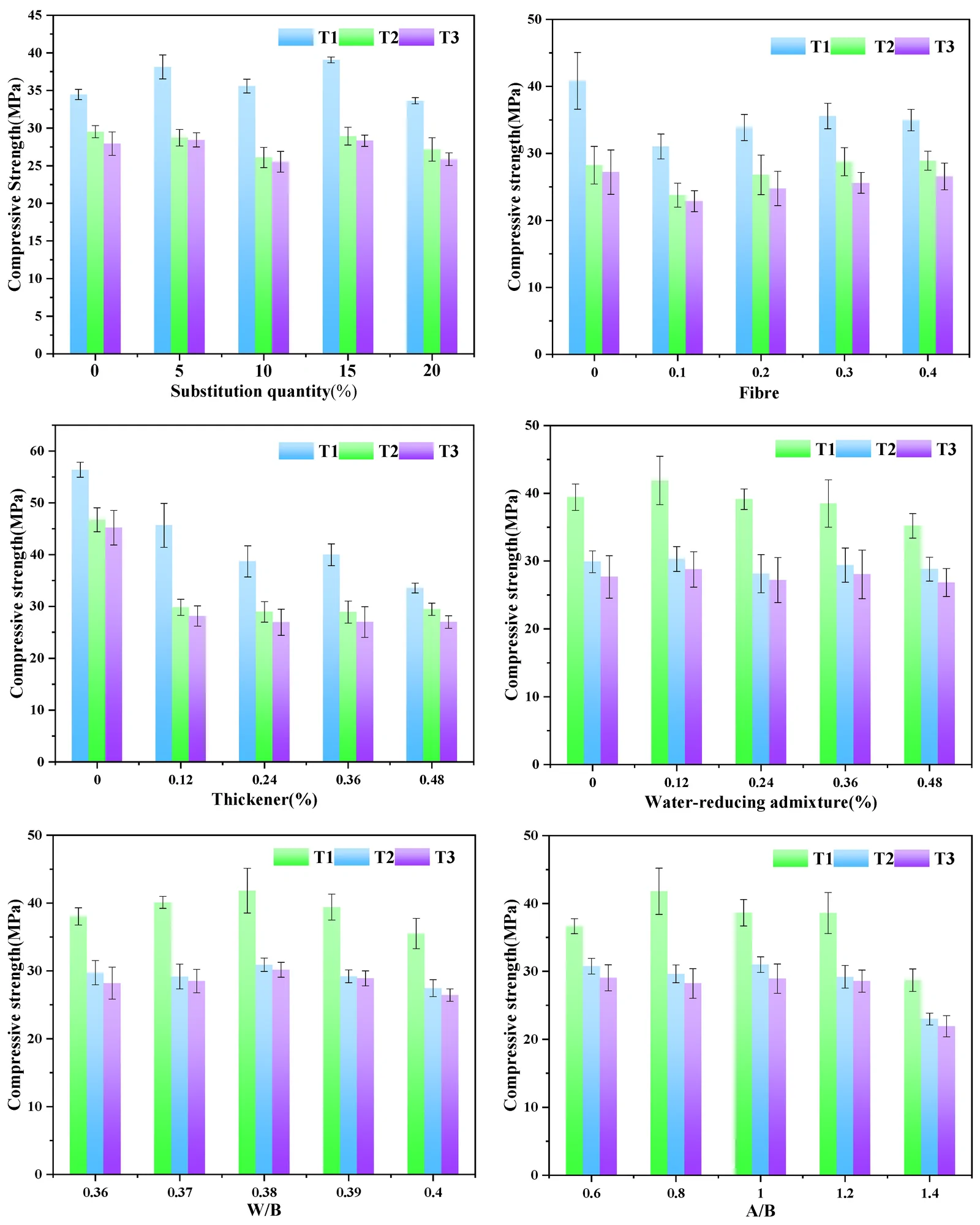
Compressive and flexural strength charts of 3DPC specimens under various parameter conditions.

The analysis of the influence of various mix design parameters on the mechanical properties of 3DPC in Figure 7 reveals an optimal substitution range for CA-RHCP. At a substitution rate of 10%, all directional strengths reach or exceed those of the reference group, primarily due to the micro-aggregate filling effect and nucleation-promoting action of CA-RHCP on hydration products. However, when the substitution rate increases to 20%, the relative insufficiency of active components leads to a generally loose hydration product structure, resulting in significant declines in all directional strengths, particularly the compressive strength, which is sensitive to defects. The water-to-binder ratio exhibits a classic negative correlation with strength, as its increase leads to higher matrix porosity and lower density. The effect of the binder-to-water ratio is nonlinear, with an optimal value (approximately 1.2 in this study). Excessively low ratios may increase shrinkage due to excess slurry, while excessively high ratios may fail to adequately encapsulate aggregates, both introducing weak interfaces that limit strength development. Additionally, while thickeners and superplasticizers optimize rheological properties, their direct impact on 28-day strength is relatively limited. However, excessive addition may interfere with hydration processes. The incorporation of fibers significantly enhances compressive strength and toughness through bridging effects, but shows limited improvement in axial load-bearing capacity. In summary, through synergistic optimization using a 10% CA-RHCP substitution rate, a water-to-binder ratio of 0.38, a binder-to-water ratio of 1.2, and appropriate amounts of thickener (0.250%) and superplasticizer (0.24%), the optimal balance of 3DPC’s comprehensive mechanical properties can be achieved while maintaining excellent printability.

#### 3.2.2. Microscopic Characteristics of 3D Printing Components

**Figure 8:**
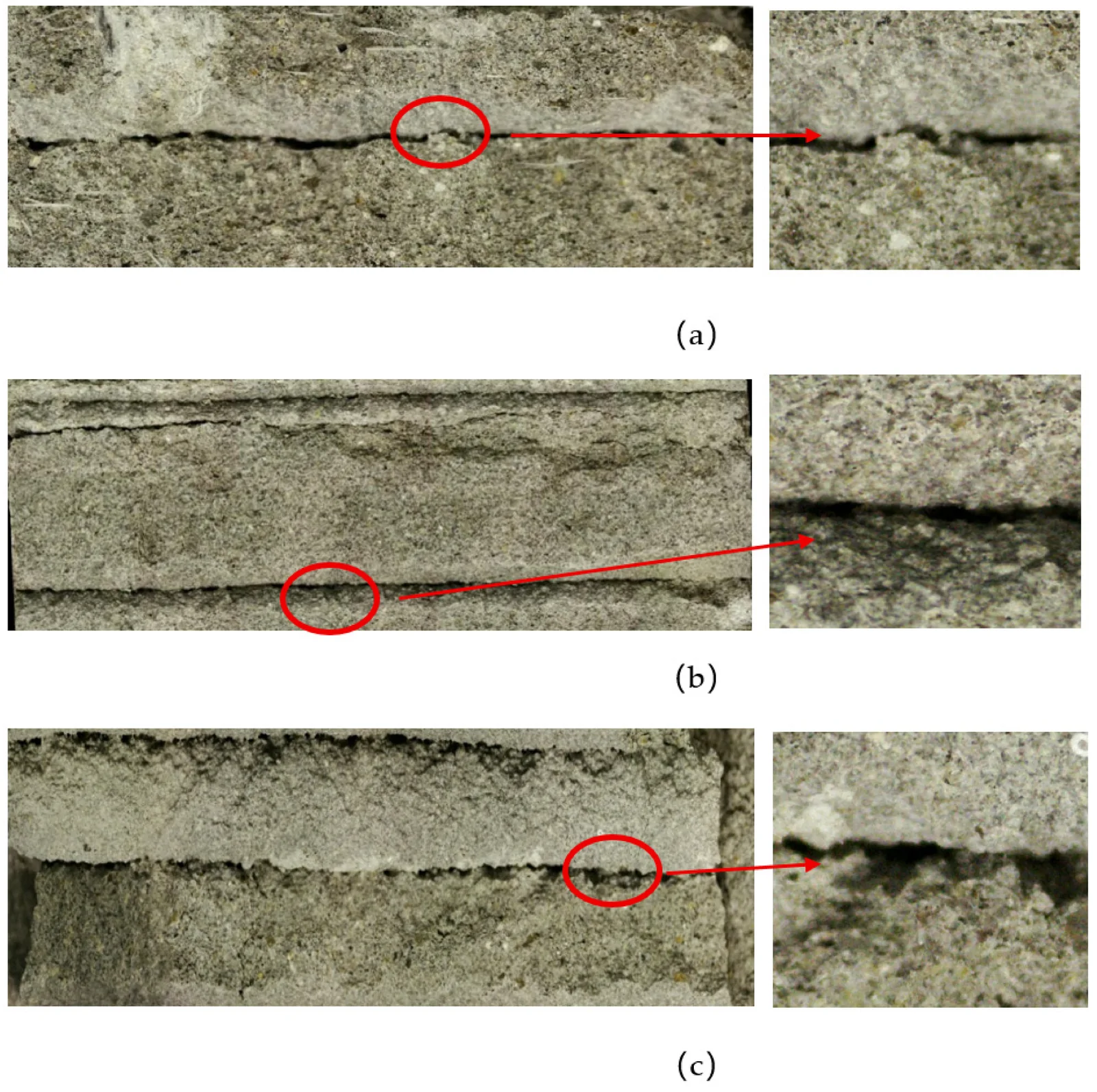
Anisotropic interlayer images under optical microscopy: (**a**) T1 direction, (**b**) T2 direction, (**c**) T3 direction.

The mechanical properties of 3D-printed components exhibit significant anisotropy, with the core weakness lying in the interlayer interfaces perpendicular to the printing direction. Optical microscopy observations of these interfaces clearly demonstrate the direct correlation between pore structure and macroscopic mechanical performance. From Figure 8, Analysis of the images reveals that the mechanical strength of specimens is closely related to the scale and distribution of interlayer pores: regions with higher strength typically display finer, more dispersed pore structures, while lower-strength areas correspond to larger, more continuous pores. Specifically, the T1 direction demonstrates the highest compressive strength, corresponding to the smallest and most densely distributed interlayer pores. Conversely, the T3 direction exhibits the lowest compressive strength, with significantly larger and more interconnected interlayer pores. This phenomenon directly confirms the inherent anisotropy characteristics of 3D-printed concrete due to its layer-by-layer accumulation process—material continuity is better within the printing plane, whereas interlayer interfaces are relatively weaker.

**Figure 9:**
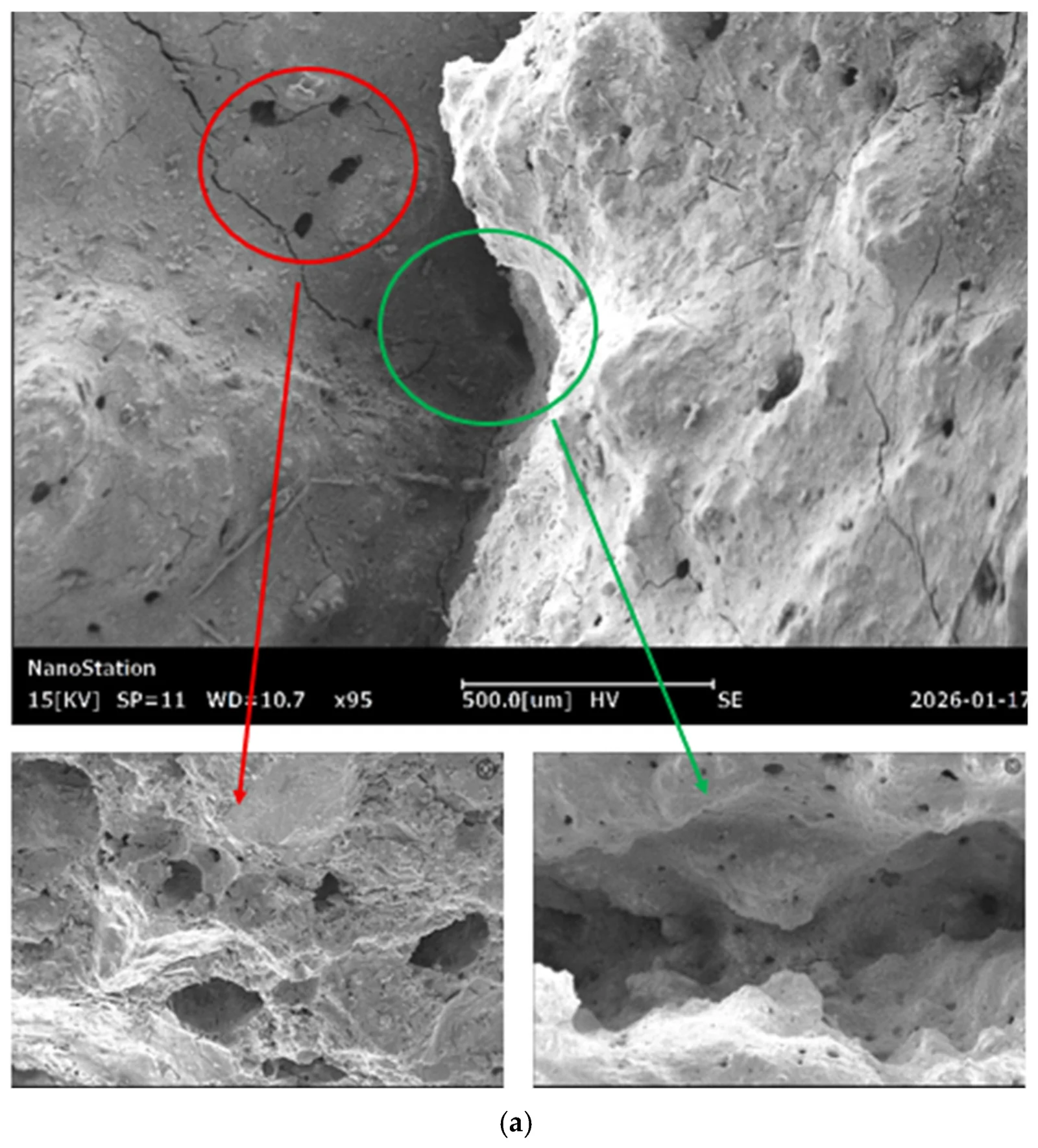

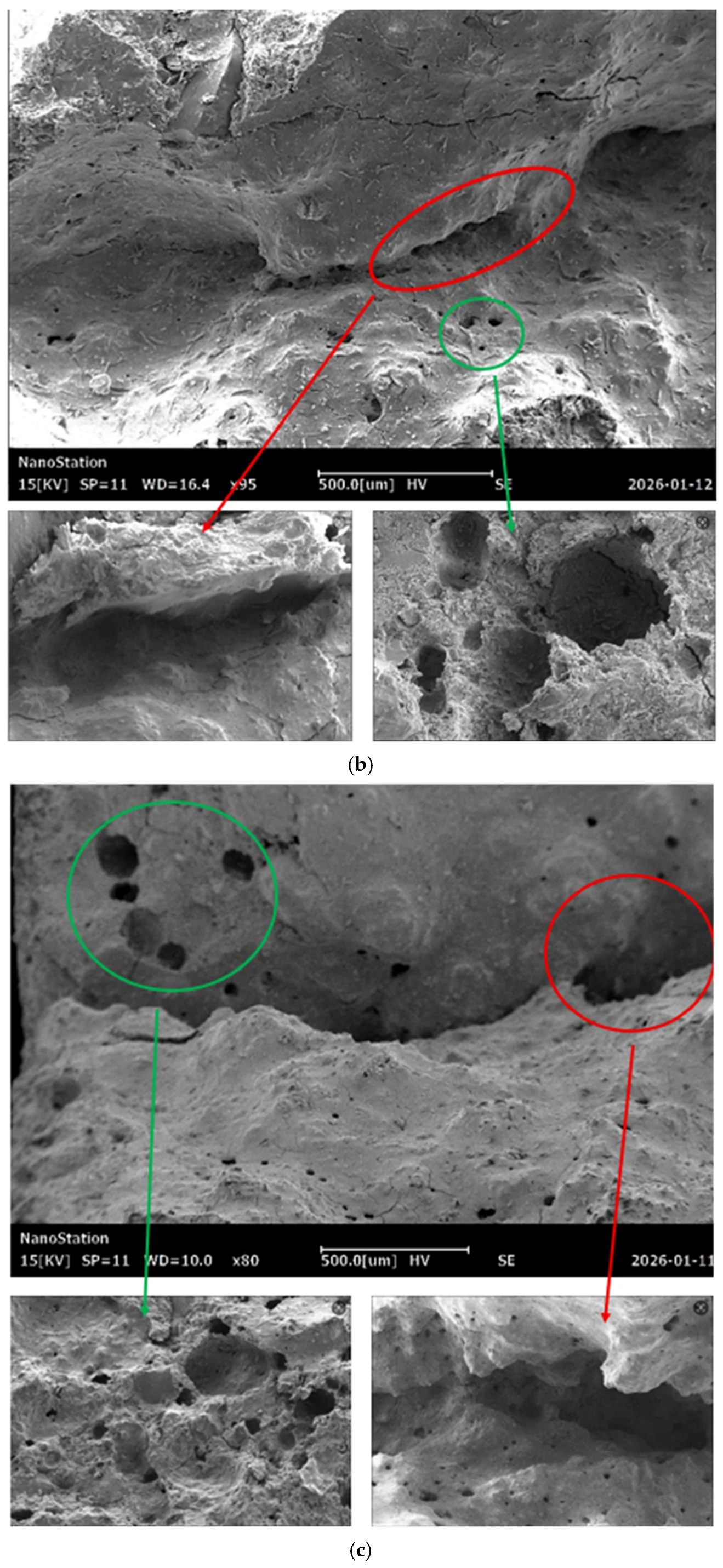
SEM images showing global and local magnification comparisons of interlayer fracture zones: (**a**) T1, (**b**) T2, (**c**) T3.

The SEM images in Figure 9, which compare the global and local magnification of interlayer fracture zones, demonstrate that the compressive strength in the T1 direction is likely higher than that in the T3 direction and slightly exceeds that in the T2 direction. This is attributed to electron microscopy observations revealing that the T3 direction is significantly smaller than the T1 direction. This difference directly stems from the layered deposition characteristics of 3D printing: when printing upward along the T3 direction, interlayer fusion is often incomplete, leading to the formation of numerous larger pores and unbonded regions, which create weak internal interfaces. Conversely, when printing upward along the T1 direction, the material deposits continuously within each layer, resulting in a denser structure with lower porosity. Consequently, under compressive stress, pores in the T3 direction tend to become stress concentration points and crack initiation sites, causing its compressive strength to be significantly lower than that in the T1 direction. This phenomenon exemplifies the typical anisotropic behavior of 3D-printed materials, where interlayer bonding dominates the mechanical properties. In Figure 9a, the upper image is a low-magnification overview of the interlayer fracture zone, with a magnification of ×95 and a scale bar of 500 μm. The lower images are high-magnification local views of the regions marked by the red and green ellipses in the upper image. The red region was magnified at ×100 with a scale bar of 500 μm, and the green region was magnified at ×97, also with a scale bar of 500 μm. The red ellipses indicate typical interlayer pores, while the green ellipses indicate unbonded or weakly bonded regions. These magnification levels allow simultaneous observation of the overall pore distribution at the interlayer interface and the local micromorphology of hydration products and bonding states. Similarly, in Figure 9b, the red region was magnified at ×90, and the green region at ×200; in Figure 9c, the red region was magnified at ×100 and the green region at ×50. All scale bars are 500 μm.

**Figure 10:**
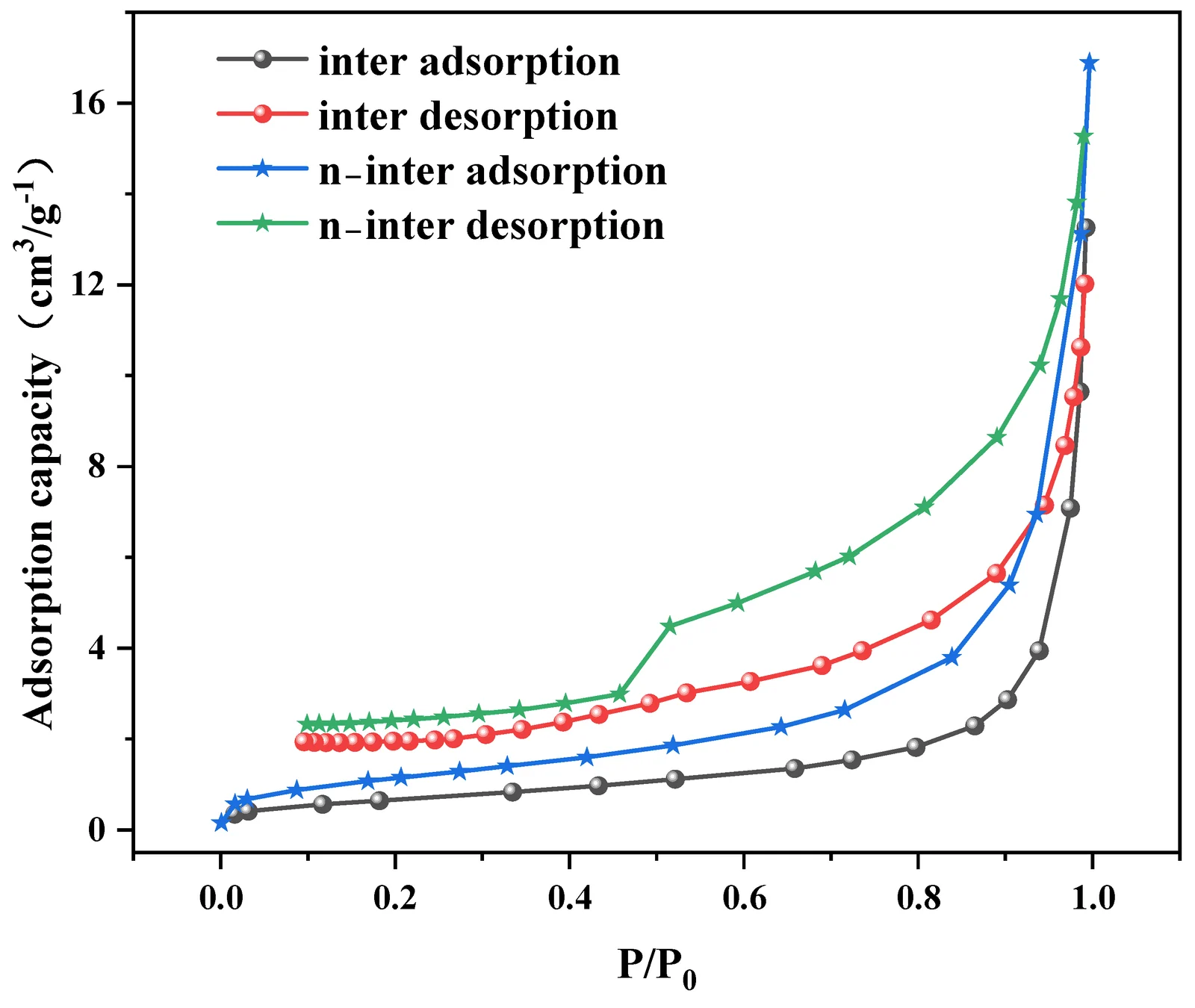
Pore size analysis of interlayer and non-interlayer test samples.

The analytical results of the pore structure in the 3D-printed concrete sample (Figure 10) reveal a significant difference in pore size distribution between the non-layered and layered regions. This microstructure-level variation reflects the printing process’s impact on the material’s internal arrangement. In the layered region, the average pore size is relatively small, and the pore distribution curve is more concentrated, indicating that a layer-to-layer contact interface has been achieved with better results in terms of density and pore dislocation under the printing conditions. This phenomenon is primarily attributed to the effective pressure exerted on the material during printing, as well as the good interfacial melting of the fluid between layers. In contrast, the non-layered region exhibits a relatively larger pore size and broader distribution range, reflecting a more fragile accumulation of materials. This may be the result of multiple factors, such as printing path layout, material flow properties, and stress distribution during the hardening phase. It should be noted that this spatial variation in the pore structure is clearly correlated with the mechanical property test results of the specimen. Since the laminated region features a more uniform and smaller porous structure with fewer internal defects, it not only helps in stress transfer and distribution but also enhances the mechanical effect of interfacial bonding and chemical adhesion, resulting in significant improvements in pressure resistance and interlayer adhesion strength in this direction.

### 3.3. Model Prediction and Analysis of Printing Strength

This study constructed and systematically evaluated a machine learning-based 3D printing concrete mechanical property prediction model, aiming to establish a quantitative mapping relationship between material composition, process parameters, and multi-directional compressive strength. Three representative algorithms, XGBoost, Random Forest, and LightGBM, were respectively applied to the prediction tasks of compressive strength in the X, Y, and Z directions. The model performance was comprehensively evaluated through mean absolute error (MAE), root mean square error (RMSE), and coefficient of determination (R^2^).

As shown in Table 2, the XGBoost model exhibited the highest prediction accuracy and stability in all directions. In the X, Y, and Z directions, the MAE was 1.44 MPa, 1.48 MPa, and 1.48 MPa, respectively, and the RMSE was 3.03 MPa, 3.04 MPa, and 3.78 MPa, respectively. The R^2^ values were all above 0.91, indicating that this model has a relatively accurate ability to capture the complex nonlinear relationships and anisotropic characteristics in the 3DPC mechanical behavior. Although the Random Forest model was slightly inferior to XGBoost, its prediction ability was still good, with an R^2^ value of 0.88. The LightGBM model had a large fluctuation in the prediction error in the Z direction, with an RMSE as high as 8.90 MPa. The example shows that its generalization ability is limited when dealing with mechanical responses dominated by inter-layer interfaces.

These error levels are competitive with, but not uniformly superior to, benchmarks reported for data-driven 3DPC strength prediction. A recent study using Gaussian process regression on a literature-extracted dataset with 21 mix features achieved R^2^ values exceeding 0.91 for anisotropic printed strengths, with RMSE below 4.1 MPa. Another study employing bootstrap-augmented XGBoost reported R^2^ up to 0.982 for direction-specific printed compressive strength, albeit on an augmented dataset whose size and augmentation strategy differ fundamentally from the 25-mixture orthogonal design used here. The present dataset is deliberately small and physically controlled—25 mixtures, 675 compressive strength data points—rather than assembled from heterogeneous literature sources. This choice trades breadth for internal consistency: the features share a common testing protocol and material provenance, but the resulting models are necessarily more constrained in generalization than models trained on aggregated multi-source data. The LightGBM model‘s large Z-direction error (MAE = 2.30 MPa, RMSE = 8.90 MPa) is therefore not simply a “worse algorithm” result; it reflects the specific difficulty of learning interlayer-dominated behavior from a dataset in which the interlayer direction carries the largest variance and the smallest absolute strength, a condition under which leaf-wise growth strategies are prone to overfitting local interface configurations. Recent work incorporating explainable machine learning—SHAP and ICE analyses—has shown that water-to-cement ratio and loading direction are among the most globally influential variables for anisotropic 3DPC strength; the feature-importance ranking obtained here, in which CA-RHCP substitution and water-to-binder ratio dominate, is consistent with that finding and provides a material-specific confirmation from an independent dataset [38].

**Figure 11:**
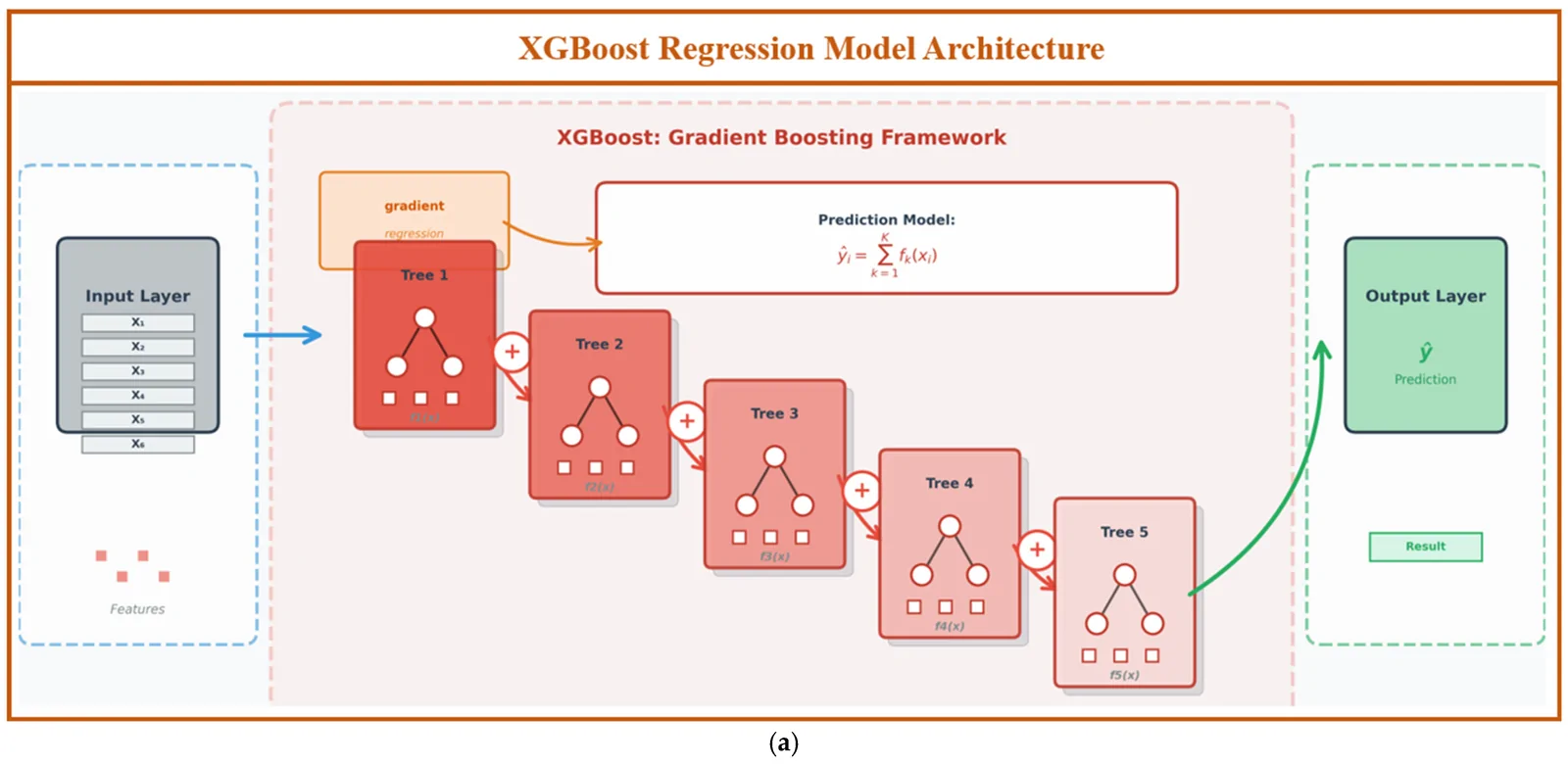

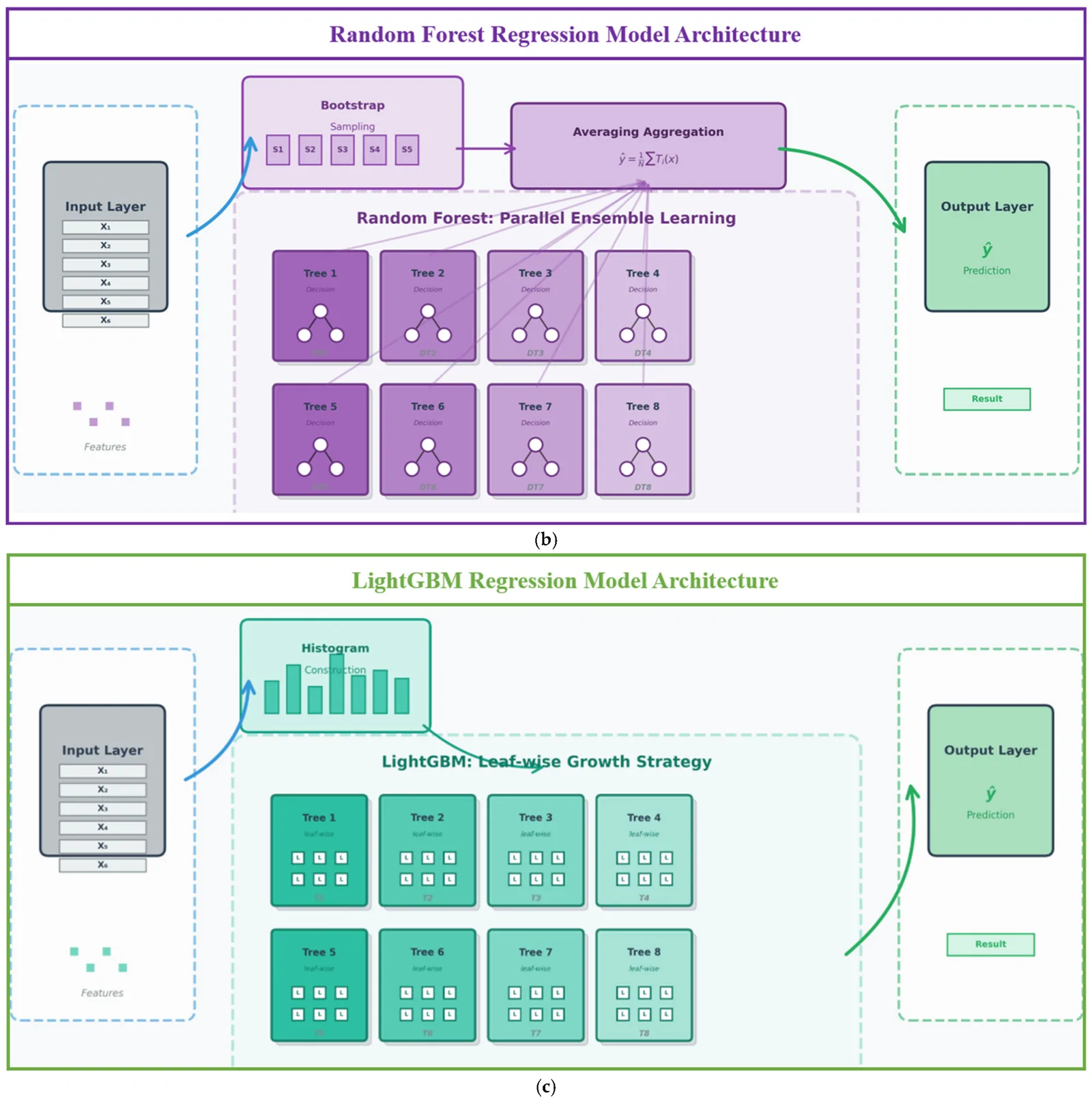
Regression Model Architecture of Various Strength Prediction Models. (**a**) XGBoost (**b**) Random Forest (**c**) LightGBM.

As shown in Figure 11, the construction processes of the three machine learning models, XGBoost, Random Forest, and LightGBM, were systematically compared. Their overall architectures followed the typical data-driven modeling pattern: Firstly, based on the material proportion parameters obtained from orthogonal experiments and their corresponding multi-directional compressive strength data, data preprocessing and feature engineering were implemented; then, the data set was divided into training and test sets, which were used for model training and validation; each model learned the complex nonlinear mapping relationship between features and strength through internal algorithms; finally, the prediction accuracy and generalization ability were comprehensively evaluated by indicators such as mean absolute error, root mean square error, and coefficient of determination. Among the three ensemble learning models, XGBoost, Random Forest, and LightGBM adopted different modeling strategies, demonstrating the evolution and optimization paths of ensemble learning in different dimensions. Random Forest adopted a parallel integration method, generating multiple independent decision trees through Bootstrap sampling, and aggregating the prediction results in an average manner. Its structure diagram shows eight parallel decision trees, embodying the “bagging” concept, and having strong anti-overfitting ability and stability. XGBoost adopted a serial gradient-boosting structure, fitting the residuals of each tree sequentially, gradually improving the prediction accuracy. Its architecture diagram labels “gradient regression”, clearly revealing the optimization mechanism based on gradients. LightGBM further optimized and improved efficiency, introducing histogram-based algorithms and leaf-wise growth strategies. In the Figure 11, each tree is represented by “L” for multiple leaf nodes, highlighting its leaf-splitting characteristic. This strategy is more effective than the traditional layer-wise growth in reducing the loss function, but it is necessary to control max_depth and num_leaves to prevent overfitting. Overall, the input layers of all three adopt the same feature dimension (X1 to X6), but there are significant differences in model structure, tree interaction methods, and parameter tuning strategies. These differences determine their performance in different datasets and tasks, and provide theoretical support for subsequent result analysis and model selection.

**Figure 12:**
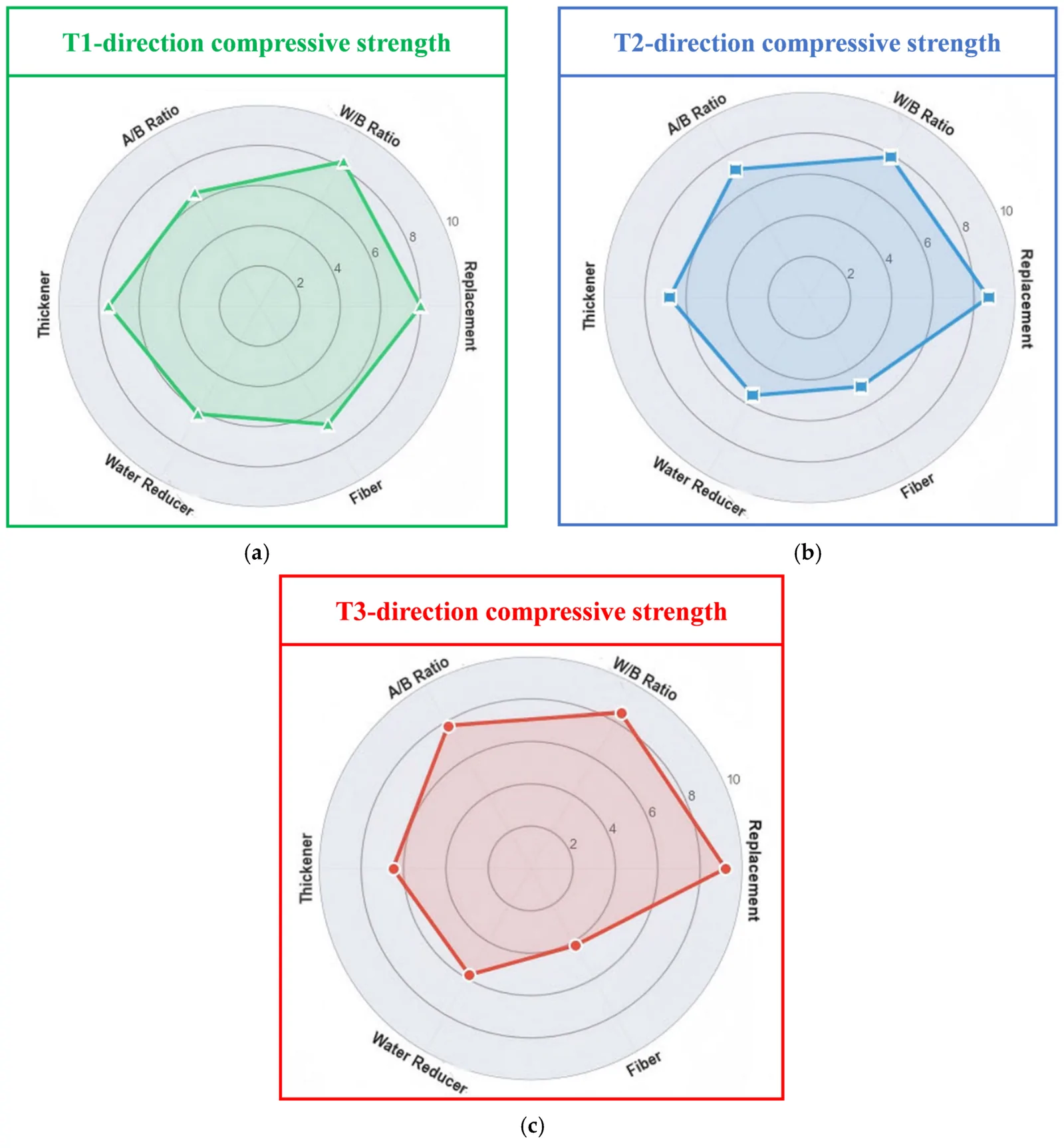
Radar chart of characteristic importance of compressive strength in different directions: (**a**) T1 (**b**) T2 (**c**) T3.

From Figure 12, The key factors influencing the strength in different directions can be further revealed through feature importance analysis. The material parameters that have the most significant impact on compressive strength are the substitution amount of CA-RHCP and the water-cement ratio. The prediction of the strength in the T1 direction relies more on rheology-related parameters such as the amount of thickener and the amount of fibers. This phenomenon is consistent with the mechanism that the strength in this direction is dominated by the interlayer bonding quality. The dosage of the water reducer has a relatively prominent effect on the strength in the T2/T3 directions. Its role in regulating the uniformity and density of the material within the printing plane can be seen as evidence that the importance of the influencing factors of compressive strength in different directions varies.

**Figure 13:**
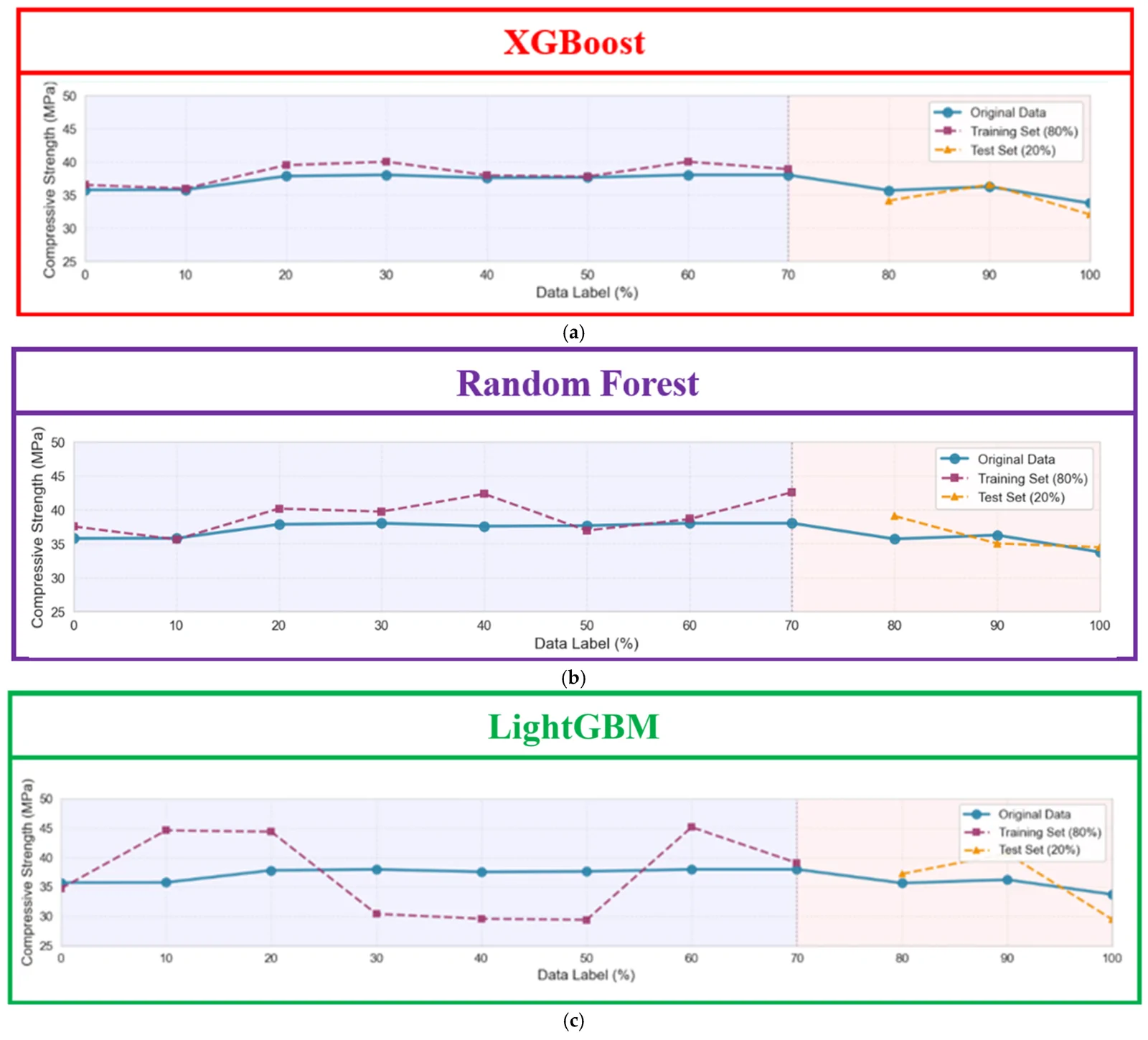
Prediction process and results of concrete compressive strength for various models. (**a**) XGBoost (**b**) Random Forest (**c**) LightGBM.

As can be seen from Figure 13, each model utilizes 80% of the training data and 20% of the test data for model training and prediction. Regarding the presentation of this complete prediction process, from data preprocessing and feature engineering to model training and evaluation, it appears as a chain-like display form; in this specific context, the advantages in fitting for multi-variable and small sample datasets are demonstrated, to some extent, reflecting the characteristics driven by the XGBoost operational mode. The reliable and efficient quantitative tool attributes corresponding to this data-driven method are presented, to some extent, demonstrating a supporting role; the promotion of precise and high-value application of construction waste in high-performance intelligent manufacturing has also been given a certain degree of impetus.

Rather than treating the observed anisotropy, the predictive models, and the study’s limitations as three separate outputs, it is more accurate to read them as three expressions of a single positioning. First, the moderate anisotropy reported here is not an isolated material property but a signature of CA-RHCP’s differential action on the bulk matrix versus the interlayer transition zone. The modified powder simultaneously alters surface chemistry (through nanoscale CaCO_3_ nucleation sites), rheology (through reduced water absorption and modified particle packing), and interfacial mechanics (through densification of the interlayer region), and it is the coupling among these three effects—rather than any one of them in isolation—that produces the observed T1 > T2 > T3 hierarchy with a relatively contained directional spread. Existing studies have not systematically decoupled these effects, and the present results therefore reframe anisotropy as a process- and interface-controlled response rather than a fixed material attribute. Second, the machine-learning results are deliberately framed as a locally valid and mechanistically interpretable prediction tool, whose value derives precisely from the constrained, physically controlled dataset rather than from a claim to universal accuracy. The comparison among XGBoost, Random Forest, and LightGBM is not presented as a ranking of algorithms in the abstract; it is used to show that interlayer-dominated variance in the Z direction imposes specific learning difficulties that are masked in the literature-aggregated datasets. Third, the limitations of this study are formulated as literature-anchored boundary conditions—single-source versus multi-source RCP, laboratory-scale versus full-scale LCA, and short-term strength versus long-term durability—rather than as generic disclaimers. Each boundary marks where the present conclusions cease to be transferable and where subsequent work must begin. Read together, these three moves distinguish the present study from work that reports anisotropy as a standalone number, ranks models by aggregate accuracy, or lists caveats without external reference points; they also clarify the conditions under which CA-RHCP-modified 3DPC can be regarded as a technically viable and environmentally defensible material route.

## 4. Conclusions

This study systematically investigated the effects of microbial acidification and carbon fixation activation-modified recycled micro-powder (CA-RHCP) on the printability and mechanical properties of 3D-printed concrete (3DPC), and constructed a performance prediction model based on machine learning, leading to the following four conclusions:

(1) The compressibility of 3D-printed concrete (3DPC) is synergistically regulated by several key parameters, including the CA-RHCP replacement rate, water-cement ratio, dosage of superplasticizer, dosage of thickener, aggregate-to-cement ratio, and fiber content. Research shows that under optimized parameter conditions, 3DPC exhibits excellent compressibility and formability. The flow diameter in the flow table test remains stable at 160–170 mm with a moderate open time. There are synergistic and trade-off relationships between these parameters. For example, increasing the amount of superplasticizer improves flowability but reduces open time, while adding a thickener has the opposite effect. Through multi-parameter optimization, an optimal balance between material compressibility and workability can be achieved, ensuring continuous printing of 20 layers (height 200 mm) without collapse.

(2) The mechanical properties of 3DPC exhibit significant anisotropy, with strength increasing in the following order: T1 direction > T2 direction > T3 direction. Factors such as the CA-RHCP replacement rate, water binder ratio, aggregate binder ratio, and fiber content collectively influence strength development. An optimal balance of anisotropic strength is achieved when the CA-RHCP replacement rate is 10%, the water binder ratio is 0.38, and the aggregate binder ratio is 1.2. Microstructural analysis reveals that pore structures between layers are a critical factor in strength across all directions: the T1 direction has the finest and most uniformly distributed pores, resulting in the highest strength; conversely, the T3 direction shows larger and more interconnected pores, leading to lower strength. Incorporation of fibers significantly enhances resistance and compressive strength, while thicker materials and superplasticizers have limited effect on 28-day strength; excessive addition may even weaken interfacial bonding.

(3) Strength prediction models based on machine learning algorithms: XGBoost, Random Forest, and LightGBM can effectively establish nonlinear relationships between material composition and multi-directional compressive strength. Among these, the XGBoost model performs best, achieving the highest forecasting accuracy: MAE < 1.5 MPa, R^2^ ≥ 0.91, making it suitable for multivariate datasets with small sample sizes. An analysis of feature importance further reveals that the CA-RHCP replacement rate and water binder ratio are the most significant factors influencing strength, while the T1 direction’s strength is more dependent on rheological parameters. This model provides a reliable quantitative tool for material design and process optimization in 3DPC, facilitating the high-quality utilization of construction waste in intelligent buildings.

(4) Several boundaries of this study merit explicit discussion in light of existing evidence. First, the single-source CA-RHCP used here means that batch-to-batch compositional variability remains unquantified; the broader RCP literature increasingly emphasizes that powder reactivity and carbonation response depend on parent concrete mineralogy and service history, and multi-source standardization is a recognized prerequisite for reproducibility. Second, the 10% replacement ceiling is a real constraint: at ≥15%, the dilution of reactive phases outweighs the nucleation and filling benefits, a threshold effect that is consistent with the broader observation that unmodified or partially activated RCP typically causes strength reduction above 10–15% replacement. Third, the laboratory-scale printing and mechanical testing leave open the question of whether the observed anisotropy hierarchy and interlayer densification persist in full-scale elements under realistic curing and loading conditions. Life-cycle assessment studies of 3D-printed structural units using construction and demolition waste have shown that the environmental benefits of 3D printing can be offset by high cement content and material transport, underscoring that laboratory-scale “green” material substitutions require system-level verification before sustainability claims can be substantiated. Fourth, the 25-mixture dataset, while internally consistent, cannot support claims of generalization to other binder systems or printing equipment; the models developed here should be treated as locally valid quantitative tools rather than universal predictors. Fifth, long-term durability—carbonation, chloride ingress, freeze–thaw, and creep—was not assessed, yet interlayer interfaces are known to act as preferential transport pathways that accelerate deterioration. Each of these boundaries does not diminish the contribution of the present work but defines the conditions under which its conclusions hold, and together they map a focused agenda for subsequent research [39].

## Figures and Tables

**Table 1:** Specific data for each design mix proportion.

Variable Name	Variable Gradient				
Replacement	0%	5%	10%	15%	20%
W/B	0.36	0.37	0.38	0.39	0.40
A/B	0.8	1.0	1.2	1.4	1.6
Thickener	0.00%	0.12%	0.24%	0.36%	0.48%
Water reducer	0.000%	0.125%	0.250%	0.375%	0.500%
Fiber	0.0%	0.1%	0.2%	0.3%	0.4%

**Table 2:** Data Table of Prediction Results for Compressive Strength Analysis of Various Models.

Model	Statistical Indicators	X	Y	Z
XGBoost	MAE (MPa)	1.44	1.48	1.48
	RMSE (MPa)	3.03	3.04	3.78
	R^2^	0.94	0.92	0.91
	MAE (MPa)	1.51	1.55	1.56
RF	RMSE (MPa)	3.28	3.32	3.96
	R^2^	0.90	0.89	0.88
	MAE (MPa)	1.49	1.37	2.30
LightGBM	RMSE (MPa)	3.07	2.71	8.90
	R^2^	0.72	0.72	0.78

## Data Availability

The original contributions presented in this study are included in the article. Further inquiries can be directed to the corresponding author.

## References

[1] Paul S.C., Van Zijl G.P., Tan M.J., Gibson I. (2018). A review of 3D concrete printing systems and materials properties: Current status and future research prospects. Rapid Prototyp. J..

[2] Buswell R.A., De Silva W.R.L., Jones S.Z., Dirrenberger J. (2018). 3D printing using concrete extrusion: A roadmap for research. Cem. Concr. Res..

[3] Lim S., Buswell R.A., Valentine P.J., Piker D., Austin S.A., De Kestelier X. (2016). Modelling curved-layered printing paths for fabricating large-scale construction components. Addit. Manuf..

[4] Zhao Z., Ji C., Xiao J., Yao L., Lin C., Ding T., Ye T. (2023). A critical review on reducing the environmental impact of 3D printing concrete: Material preparation, construction process and structure level. Constr. Build. Mater..

[5] Panda B., Paul S.C., Hui L.J., Tay Y.W.D., Tan M.J. (2017). Additive manufacturing of geopolymer for sustainable built environment. J. Clean. Prod..

[6] Chen Y., Jansen K., Zhang H., Rodriguez C.R., Gan Y., Çopuroğlu O., Schlangen E. (2020). Effect of printing parameters on interlayer bond strength of 3D printed limestone-calcined clay-based cementitious materials: An experimental and numerical study. Constr. Build. Mater..

[7] Le T.T., Austin S.A., Lim S., Buswell R.A., Gibb A.G.F., Thorpe T. (2012). Mix design and fresh properties for high-performance printing concrete. Mater. Struct..

[8] Paul S.C., Tay Y.W.D., Panda B., Tan M.J. (2018). Fresh and hardened properties of 3D printable cementitious materials for building and construction. Arch. Civ. Mech. Eng..

[9] Huang B., Wang X., Kua H., Geng Y., Bleischwitz R., Ren J. (2018). Construction and demolition waste management in China through the 3R principle. Resour. Conserv. Recycl..

[10] Zhang J., Shi C., Li Y., Pan X., Poon C.-S., Xie Z. (2015). Performance Enhancement of Recycled Concrete Aggregates through Carbonation. J. Mater. Civ. Eng..

[11] Zhao Y., Cui K., He J., Zheng Y., Shen P., Poon C.S., Peng G., Guo R., Xia D. (2024). Highly reactive carbonated recycled concrete fines prepared via mechanochemical carbonation: Influence on the early performance of cement composites. Cem. Concr. Compos..

[12] Wang L., Wang J., Qian X., Chen P., Xu Y., Guo J. (2017). An environmentally friendly method to improve the quality of recycled concrete aggregates. Constr. Build. Mater..

[13] Abriak Y., Chu D.C., Maherzi W., Benzerzour M., Rivard P. (2023). Influence of fine recycled concrete aggregates use on the hydration kinetics and mechanical–microstructural properties of hydrated cement: Experimental and numerical approaches. Constr. Build. Mater..

[14] Zhang D., Zhang S., Huang B., Yang Q., Li J. (2022). Comparison of mechanical, chemical, and thermal activation methods on the utilisation of recycled concrete powder from construction and demolition waste. J. Build. Eng..

[15] Ashraf W. (2016). Carbonation of cement-based materials: Challenges and opportunities. Constr. Build. Mater..

[16] Reiter L., Wangler T., Roussel N., Flatt R.J. (2018). The role of early age structural build-up in digital fabrication with concrete. Cem. Concr. Res..

[17] Moeini M.A., Hosseinpoor M., Yahia A. (2022). 3D printing of cement-based materials with adapted buildability. Constr. Build. Mater..

[18] Márquez Á., Varela H., Barluenga G. (2024). Rheology and early age evaluation of 3D printable cement-limestone filler pastes with nanoclays and methylcellulose. Constr. Build. Mater..

[19] Saruhan V., Keskinateş M., Felekoğlu B. (2022). A comprehensive review on fresh state rheological properties of extrusion mortars designed for 3D printing applications. Constr. Build. Mater..

[20] Lim S., Buswell R., Le T., Austin S., Gibb A., Thorpe T. (2011). Developments in construction-scale additive manufacturing processes. Autom. Constr..

[21] Khan S.A., Jassim M., Ilcan H., Sahin O., Bayer I.R., Sahmaran M., Koc M. (2023). 3D printing of circular materials: Comparative environmental analysis of materials and construction techniques. Case Stud. Constr. Mater..

[22] Krishnaraja A., Guru K. (2021). 3D Printing Concrete: A Review. IOP Conf. Ser. Mater. Sci. Eng..

[23] Khosravani M.R., Haghighi A. (2022). Large-Scale Automated Additive Construction: Overview, Robotic Solutions, Sustainability, and Future Prospect. Sustainability.

[24] To Q.B., Pham K.V.A., Lee G., Shin M., Shin D.W., Lee K. (2024). Experimental and FEM evaluation of the influence of interlayer bonding strength in 3D printed concrete members under compressive and flexural loadings. J. Build. Eng..

[25] Wang C., Chen B., Vo T.L., Rezania M. (2023). Mechanical anisotropy, rheology and carbon footprint of 3D printable concrete: A review. J. Build. Eng..

[26] Baduge S.K., Navaratnam S., Abu-Zidan Y., McCormack T., Nguyen K., Mendis P., Zhang G., Aye L. (2021). Improving performance of additive manufactured (3D printed) concrete: A review on material mix design, processing, interlayer bonding, and reinforcing methods. Structures.

[27] Taborda-Llano I., Hoyos-Montilla A.A., Asensio E., Guerrero A.M., Tobón J.I. (2026). Influence of the construction process parameters on the mechanical performance and durability of 3DPC (3DPC): A systematic review. J. Build. Eng..

[28] Paudel S. (2023). Investigation of modelling approaches to study the structural performance of 3D printed plain wall under uniform axial compression. Adv. Struct. Eng..

[29] Wolfs R., Bos F., Salet T. (2019). Hardened properties of 3DPC: The influence of process parameters on interlayer adhesion. Cem. Concr. Res..

[30] Cavalcante T.C., Filho R.D.T., Reales O.A.M. (2025). Rheological and Environmental Implications of Recycled Concrete Powder as Filler in Concrete 3D Printing. Buildings.

[31] Abdellatief M., Saqr A.M. (2026). Machine learning prediction of compressive strength in 3d printed fiber reinforced concrete using support vector regression and artificial neural networks with shapley additive explanations. Sci. Rep..

[32] (2020). Standard Test Method for Flow of Hydraulic Cement Mortar.

[33] (2009). Standard for Test Method of Basic Properties of Construction Mortar.

[34] Izadgoshasb H., Kandiri A., Shakor P., Laghi V., Gasparini G. (2021). Predicting Compressive Strength of 3D Printed Mortar in Structural Members Using Machine Learning. Appl. Sci..

[35] Arif M., Jan F., Rezzoug A., Afridi M.A., Luqman M., Khan W.A., Kujawa M., Alabduljabbar H., Khan M. (2024). Data-driven models for predicting compressive strength of 3D-printed fiber-reinforced concrete using interpretable machine learning algorithms. Case Stud. Constr. Mater..

[36] Duarte G., Duarte J.P., Memari A., Brown N., Gevaudan J.P. (2023). Towards a model for structural performance in concrete printing based on buildability and toolpath design. J. Build. Eng..

[37] Du H., Khan M., McNally C., Ozer E. (2026). Advancements and challenges in 3D concrete printing: An integrated insight into material formulation, structural performance and printing strategies for sustainable future. J. Sustain. Cem.-Based Mater..

[38] Malik U.J., Riaz R.D., Rehman S.U., Usman M., Riaz R.E., Hamza R. (2024). Advancing mix design prediction in 3D printed concrete: Predicting anisotropic compressive strength and slump flow. Case Stud. Constr. Mater..

[39] Wang Y., Sun J., Wang X., Huang B., Xu S., Shang J., Li S., Song C., Wang D. (2025). Environmental and economic evaluation of a prefabricated 3D-printed structural units using recycled aggregates from construction and demolition waste: A case study in China. Energy Build..

